# Two New Altenusin/Thiazole Hybrids and a New Benzothiazole Derivative from the Marine Sponge-Derived Fungus *Alternaria* sp. SCSIOS02F49

**DOI:** 10.3390/molecules23112844

**Published:** 2018-11-01

**Authors:** Yaping Chen, Ruyan Chen, Jinhuai Xu, Yongqi Tian, Jiangping Xu, Yonghong Liu

**Affiliations:** 1 School of Pharmaceutical Sciences, Southern Medical University, Guangzhou 510515, China; zlchenyaping@126.com; 2 College of Biological Science and Technology, Fuzhou University, Fuzhou 350116, China; 13107668079@163.com (R.C.); xujinhuai@yahoo.com (J.X.); 3 CAS Key Laboratory of Tropical Marine Bio-Resources and Ecology/Guangdong Key Laboratory of Marine Materia Medica/RNAM Center for Marine Microbiology, South China Sea Institute of Oceanology, Chinese Academy of Sciences, Guangzhou 510301, China

**Keywords:** sponge-derived fungus, *Alternaria* sp. SCSIOS02F49, altenusin/thiazole hybrids, benzothiazole derivative

## Abstract

Two novel altenusin-thiazole hybrids named altenusinoides A and B (**1** and **2**), a new benzothiazole derivative (**3**), and three known altenusin derivatives (**4**–**6**) have been obtained from the solid culture of the marine sponge-derived fungal strain, *Alternaria* sp. SCSIOS02F49. The structures of these new compounds were characterized by NMR, HRESIMS, and X-ray single crystal analysis. Compounds **1** and **2** possess an unusual altenusin-thiazole-fused skeleton core (6/6/5), and compound **3** represents the first benzothiazole derivative from fungi. Compounds **4** and **5** showed significant DPPH free-radical-scavenging activities with the prominent IC_50_ values of 10.7 ± 0.09 μM and 100.6 ± 0.025 μM, respectively. Additionally, compound **5** exhibited COX-2 inhibitory activity with an IC_50_ value of 9.5 ± 0.08 μM.

## 1. Introduction

Endophytic fungi have been proven to be an outstanding source of biologically active compounds with promising medicinal and agricultural applications [1]. Among them, the genus, *Alternaria*, is a prominent representative, although it is usually known as a plant pathogen [2]. Altenusin is the characteristic metabolite of *Alternaria* sp., with a biphenyl skeleton, and has been reported to have antioxidant, antimicrobial, cytotoxic, biotin protein ligase inhibitory, FXR agonist, and tau protein aggregation inhibitory activities [3,4,5,6,7,8,9]. To date, more than 15 altenusin derivatives have been discovered from fungal origins. They originate from a polyketide chain and form various skeletons, including dicyclic: Altenusin (6/6); alternariphent A1 (6/5); tricyclic: Alternariols and altenunenes (6/6/6); rubralactones (6/6/5); alterlactones (6/7/6); talaroflavones (6/5/5); and tetracyclic: Xinshengin (6/6/6/5) [10,11].

**Figure 1:**
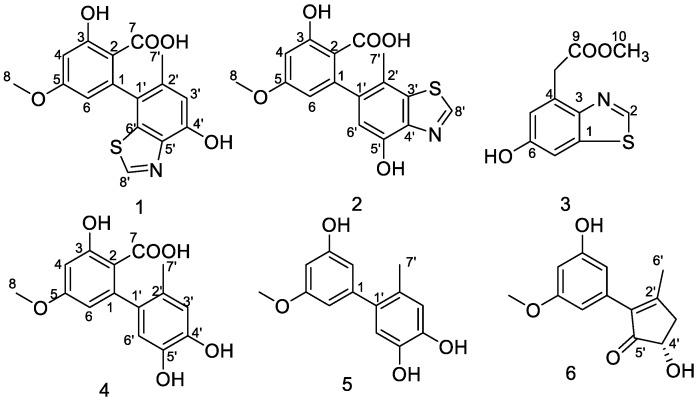
Structure of compounds **1**–**6**.

In our ongoing search for new compounds with bioactivity from marine sponge-derived fungi, a strain of *Alternaria* sp., SCSIOS02F49, isolated from a sponge, *Callyspongia* sp., was subjected to chemical study because its extracts showed an interesting HPLC and MS profile. Further isolation yielded three new compounds: Altenusinoides A (**1**), altenusinoides B (**2**), and methyl 2-(6-hydroxybenzothiazol-4-yl) acetate (**3**); along with three known compounds: Altenusin (**4**), 5′-methoxy-6-methyl-biphenyl-3,4,3′-triol (**5**), and (*S*)-alternariphent A1 (**6**) (Figure 1). Interestingly, altenusinoides A (**1**) and B (**2**) are altenusin/thiazole hybrids (6/6/5) are rarely found in nature. DPPH free-radical-scavenging activities and COX-2 inhibitory activities of these compounds were individually evaluated. Herein, we describe the isolation, structural elucidation, and bioactivity screening of these metabolites **1**–**6** from *Alternaria* sp. SCSIOS02F49. 

## 2. Results and Discussion

### 2.1. Structural Elucidation

**Figure 2:**
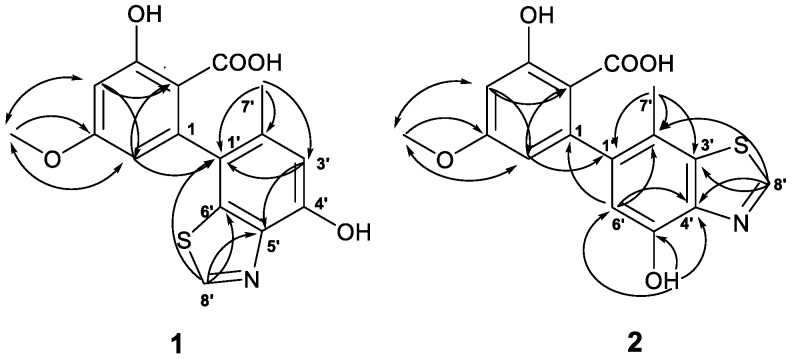
Key HMBC (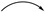) and 1D NOESY (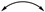) correlations of compounds **1** and **2**.

Compound **1** was obtained as white powder. The molecular formula, C_16_H_13_NO_5_S, was determined by its HRESIMS at *m*/*z* 332.0585 [M + H]^+^, indicating 11 degrees of unsaturation. The ^1^H NMR spectrum (500 MHz, DMSO-*d*_6_) showed the presence of one methyl [δ_H_ 2.06 (s, H_3_-7′)] and one methoxy, [δ_H_ 3.80 (s, H_3_-8)], four olefinic protons [δ_H_ 6.56, (d, *J* = 2.4 Hz, H-4), δ_H_ 6.24 (d, *J* = 2.4 Hz, H-6), δ_H_ 6.78 (s, H-3′), δ_H_ 9.02 (s, H-8′)], and two exchangeable protons (δ_H_ 10.11; 11.96). Analysis of the ^13^C NMR, DEPT, and HSQC spectroscopic data of **1** revealed 16 carbon signals, involving one methyl (δ_C_ 19.6, CH_3_-7′) and one methoxy (δ_C_ 55.6, CH_3_-8), four olefinic methines (δ_C_ 100.7, CH-4; δ_C_ 109.2, CH-6; δ_C_ 113.3, CH-3′; δ_C_ 152.0, CH-8′), three oxygenated quaternary carbons (δ_C_ 163.4, C-3; δ_C_ 163.3, C-5; δ_C_ 150.1, C-4′), one carboxyl carbon (δ_C_ 171.6, C-7), and six olefinic quaternary carbons (Table 1). These signals were closely related to those of altenusin (**4**), except for an additional special methine (δ_H_/δ_C_ 9.02/152.0) in **1**. By combining NMR and MS, it was easy to speculate that **1** is an analtenusin/thiazole hybrid. The location of the thiazole ring was determined by HMBC correlation. First of all, the C-7′ (δ_C_ 19.6) was located at C-2′ (δ_C_ 132.9) by the evidence of HMBC correlation of H_3_-7′ (δ_H_ 2.07) to C-1′ (δ_C_ 126.0)/C-2′/C-3′ (δ_C_ 113.3). In addition, the HMBC correlations from H-3′ (δ_H_ 6.78) to C-1′/C-4′ (150.1) and H-8′ (δ_H_ 9.02) to C-1′/C-5′ (δ_C_ 140.3)/C-6′ (δ_C_ 136.1) indicated altenusin was fused by a thiazole ring at C-5′, 6′. However, the chemical shifts of C-3 (δ_C_ 163.4) and C-5 (δ_C_ 163.3) were close to each other, so that it was not possible to unambiguously attribute an HMBC interaction, suggesting the COOH, OH, and OMe at C-2, C-3, and C-6. Therefore, selective 1D NOESY experiments were performed in CD_3_OD-*d*_4_ (Figures S9 and S10). Irradiation of OMe (δ_H_ 3.80) led to a significant enhancement of the signals of H-4 (δ_H_ 6.47) and H-6 (δ_H_ 6.17), thus confirming the OMe to be at C-5. Then, the HMBC correlation from H-6 to C-2 (δ_C_ 106.8) suggested the COOH at C-2. Finally, compound **1** was elucidated and named altenusinoide A (Figure 2). 

The molecular formula of compound **2** is C_16_H_13_NO_5_S, which was determined by HRESIMS (*m*/*z* 332.0585 [M + H]^+^). The UV and 1D NMR data ensured that compound **2** is an isomer of compound **1**, and their main difference was the location position of the thiazole ring (Table 1). The HMBC correlations of H_3_-7′ (δ_H_ 2.13) to C-1′ (δ_C_ 139.0)/C-2′ (δ_C_ 118.1)/C-3′ (δ_C_ 135.8), H-8′ (δ_H_ 9.20) to C-3′/C-4′ (δ_C_ 141.4), and H-6′ (δ_H_ 6.64) to C-1 (δ_C_ 144.0)/C-2′/C-4′/C-5′ (δ_C_ 148.6) undoubtedly showed that altenusin was fused by the thiazole ring at C-3′, 4′. Furthermore, the connection of a hydroxyl to C-5′ was provided by the HMBC correlations of OH-5′ (δ_H_ 10.04) to C-4′/C-5′/C-6′. The COOH, OH, and OMe at C-2, C-3, and C-6 was also decided by the selective 1D NOESY experiments in CD_3_OD-*d*_4_ (Figures S15 and S16). On the basis of these data, the structure of **2** was elucidated, and named altenusinoide B (Figure 2).

With its high steric hindrance at the central biaryl axis, compounds **1** and **2** should show the phenomenon of axial chirality. However, they did not show any optical rotation and CD effects in methanol, probably because they are both racemates. HPLC splitting of the two racemates on a chiral phase (Chiralpak IC) failed, which means that they are both 1:1 mixtures of rapidly interconverting atropo-diastereomeric.

**Figure 3:**
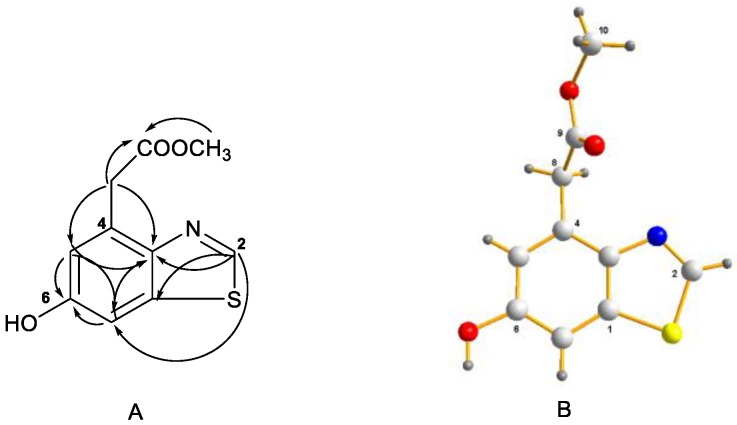
(**A**) Key HMBC correlations of compound **3**; (**B**) ORTEP diagram of compound **3**.

Compound **3** was isolated as a colorless crystal and its molecular formula, C_10_H_9_NO_3_S, was determined by HRESIMS at *m*/*z* 224.0378 [M + H]^+^ (calcd. 224.0376), requiring seven degrees of unsaturation. Comprehensive analysis of the ^1^H, ^13^C NMR, and HSQC spectra (Table 2) revealed the presence of one methylene [δ_H_/δ_C_ 4.07 (s)/36.8], one methoxy [δ_H_/δ_C_ 3.59 (s)/51.7], three olefinic methines [δ_H_/δ_C_ 9.05 (s)/151.7, 6.91 (d, *J* = 1.5 Hz)/116.8, 7.34 (d, *J* = 1.5 Hz)/105.5], five quaternary carbons (δ_C_ 145.8, 129.6, 155.4, 134.8, 171.2), and one exchangeable proton (δ_H_ 9.86). These signals were closely related to those of benzothiazole, except for an additional hydroxy unit at C-6 and methyl acetate unit at C-4. This assumption was evidenced by the key HMBC correlations and single crystal X-ray crystallographic analysis (Figure 3). Therefore, compound **3** was elucidated to be methyl 2-(6-hydroxybenzothiazol-4-yl) acetate.

By comparison of their NMR data (Table 1 and Table S2) with the reported information in the literatures, three known compounds were identified as altenusin (**4**) [12], 5′-methoxy-6-methyl-biphenyl-3,4,3′-triol (**5**) [9], and (S)-alternariphent A1 (**6**). The stereochemistry of C-4′ of **6** was established as S by the same sign of the optical rotation, optical rotation of **6**: ${\left[\alpha \right]}_{D}^{25}$ = 5.1 (c = 0.01, MeOH), while the optical rotation of (S)-alternariphent A1: ${\left[\alpha \right]}_{D}^{25}$ = 3.1 (c = 0.1, MeOH) [13,14]. 

### 2.2. Biological Activities

All compounds were evaluated for DPPH free-radical-scavenging activity and COX-2 inhibitory activity. As a result, compounds **4** and **5** showed DPPH free-radical-scavenging activity with the prominent IC_50_ values of 10.7 ± 0.09 μM and 100.6 ± 0.025 μM, respectively. Additionally, compound **5** also exhibited COX-2 inhibitory activity with the IC_50_ value of 9.5 ± 0.08 μM (Table 3). In fact, previous study has reported that compounds **4** and **5** exhibited strong radical scavenging activity in comparison with vitamin C [9].

## 3. Materials and Methods

### 3.1. General Experimental Procedures

The NMR spectra were recorded on a Bruker AC 500 NMR (Bruker, Fällanden, Switzerland) spectrometer with TMS as an internal standard. HRESIMS data were measured on a Bruker micro TOF-QII mass spectrometer (Bruker, Fällanden, Switzerland). UV spectra were recorded on a Shimadzu UV-2600 UV-Vis spectrophotometer (Shimadzu, Kyoto, Japan). X-ray diffraction intensity data were collected on a CrysAlis PRO charge-coupled device (CCD) area detector diffractometer with graphite monochromated Cu Kα radiation (λ = 1.54178 Å). Semi-preparative reversed-phase HPLC (RP-HPLC) was performed on a YMC-Pack Pro C18 RS column (5 μm, 250 × 10 mm id; YMC, Kyoto, Japan) with an Agilent 1260 (Agilent, Santa Clara, CA, USA) separation module equipped with a photodiode array (PDA) detector. Silica gel, GF254, used for TLC were supplied by the Qingdao Marine Chemical Factory (Qingdao, China). Sephadex LH-20 gel (GE Healthcare, Uppsala, Sweden) was used. Spots were detected on TLC under UV light or by heating by spraying with 12% H_2_SO_4_ in H_2_O. 

### 3.2. Fungal Materials

Fungal strain, SCSIO XWS02F49, was isolated from a sponge, *Callyspongia* sp., which was collected from the sea area near Xuwen County, Guangdong Province, China in August, 2013. The producing strain was stored on MB agar (malt extract 15 g, sea salt 10 g, agar 15 g, H_2_O 1 L, pH 7.4−7.8) slants at 4 °C and deposited at CAS Key Laboratory of Tropical Marine Bio-resources and Ecology. 

### 3.3. ITS Region Sequence and Phylogenetic Analysis

After the addition of liquid nitrogen, the mycelia of strain SCSIO XWS02F49, which was cultured in Sabouraud’s Dextrose Broth (containing 40 g of dextrose, 10 g of peptone, 2.5 g of NaCl, and 1000 mL of distilled water, pH 5.6), were sampled and powdered in a mixer mill. According to the manufacturer’s protocol, DNA was separated by the Hpure Fungal DNA Kit (Genebase Bioscience Co., GuangZhou, China). The ITS region of strain SCSIO XWS02F49 was enlarged by polymerase chain reaction with the primer pair, ITS1–ITS4. An ATIAN gel mini purification kit (TianGen Biotech, Beijing, China) was used to purify the amplified product. Pure product of PCR together with the primer, ITS1, was submitted to a commercial service (ShangHai Majorbio Bio-pharm Technology Co., Ltd., Shanghai, China) for sequencing. The sequence of the derived ITS region, which was run by BLAST-Algorithmus, were compared against the GenBank database (NCBI). Similarity analysis was processed by the ClustalW program. The nucleotide sequence has been deposited in GenBank with the accession number, KU361224.

### 3.4. Fermentation and Extraction

The seed medium (malt extract: 15 g, sea salt: 10 g, distilled water: 1000 mL, pH 7.4–7.8) was inoculated with the strain, SCSIOF49, and incubated at 25 °C for 72 h on a rotating shaker (170 rpm). The mass fermentation of this fungus was carried out using solid rice medium (rice: 200 g, sea salt: 2.5 g, distilled water: 180 mL) in 1000 mL flasks (×40). Every flask was inoculated with 10 mL of seed solution. These flasks were incubated at 25 °C under normal day night cycle. After 30 days, cultures were harvested and were soaked in acetone (500 mL/flask), mashed into small pieces, and sonicated for 15 min. Then, the acetone was evaporated under reduced pressure to afford an aqueous solution, which was extracted with EtOAc three times. At the same time, the rice residue was extracted with EtOAc to give another EtOAc solution. Both of the EtOAc solutions were combined and concentrated under reduced pressure to afford a crude extract. The crude extract was partitioned between petroleum ether and 90% aqueous MeOH to obtain a brown extract (57.0 g). 

The extract was subjected to silica gel column chromatography (CC) eluting with a PE/EtOAc mixed solvent system in a gradient eluent (*v*/*v*, 50:1, 30:1, 20:1, 10:1, 5:1, 1:1, 0:1) to obtain 7 fractions (fractions 1–7) on the basis of TLC profiles. Fr-3 (700.6 mg) was subjected to ODS chromatography eluting with MeOH/H_2_O in a gradient eluent (1:9−10:0, *v*/*v*), further purified by (SP-RP) HPLC eluting with CH_3_CN-H_2_O (38:62) to afford **1** (5.5 mg), and **2** (3.6 mg). Fr-5 (1.6 g) was subjected to Sephadex LH-20 (MeOH) to give 4 sub fractions (fr. 5.1–5.4). Fr. 5.3 (400 mg) was further purified by (SP-RP) HPLC eluting with CH_3_CN-H_2_O (28:72) to afford **3** (3.9 mg), **4** (9.8 mg), and **5** (11.3 mg). **6** (4.9 mg) was purified from Fr. 5.1 (123.5 mg) by (SP-RP) HPLC with 90% MeCN.

*Altenusinoides A* (**1**): White powder; UV (MeOH) *λ*_max_ (log ε) 204 (4.13), 302 (3.28); IR (KBr) *ν*_max_ 3320, 2935, 2750, 1578 cm^−1^. ^1^H and ^13^C NMR data, as shown in Table 1; HR-ESI-MS *m*/*z* 332.0585 [M + H]^+^ (calcd. for C_16_H_14_NO_5_S 332.0587), 354.0404 [M + Na]^+^ (calcd. for C_16_H_13_NNaO_5_S 354.0407).

*Altenusinoides B* (**2**): White powder; UV (MeOH) *λ*_max_ (log ε) 204 (4.01), 302 (2.96); IR (KBr) *ν*_max_ 3317, 2947, 2752, 1630 cm^−1^. ^1^H and ^13^C NMR data, as shown in Table 1; HR-ESI-MS *m*/*z* 332.0585 [M + H]^+^ (calcd. for C_16_H_14_NO_5_S 332.0587), 354.0407 [M + Na]^+^ (calcd. for C_16_H_13_NNaO_5_S 354.0407).

*Methyl 2-(6-hydroxybenzothiazol-4-yl) acetate* (**3**): Colorless crystals (MeOH); ^1^H and ^13^C NMR data, as shown in Table 2; the structure of **3** has been deposited in the Cambridge Crystallographic Data Centre as supplementary publication number, CCDC 1502323; HR-ESI-MS *m*/*z* 224.0378 [M + H]^+^ (calcd. for C_10_H_10_NO_3_S 224.0376), 246.0199 [M + Na]^+^ (calcd. for C_10_H_9_NNaO_3_S 246.0195).

### 3.5. Bioassay Protocols

#### 3.5.1. DPPH Radical Scavenging Activity

The DPPH (2,2-diphenyl-1-picrylhydrazyl) radical scavenging ability of the sample was conducted based on the method described by Kumagai et al. [15] with minor modifications. The reaction mixture was with DPPH (0.1 mM, dissolved in ethanol) and various concentrations of sample in equal volumes, which was incubated at room temperature for 30 min in the dark. The absorbance of the mixture was measured at 517 nm using UV (UV-2600, UNICO Instrument Co. Ltd., Shanghai, China). The final concentrations of each compound in the wells were confined as 1000, 333, 111, 37.0, 12.3, 4.11, 1.37, and 0.46 μM. The ethanol and butylated hydroxytoluene (BHT), instead of samples, were used for a blank and positive control, respectively. Each assay was carried out in triplicates. IC_50_, the concentration of elimination of 50% DPPH radical scavenging activity, was measured by non-liner regression. 

#### 3.5.2. COX-2 Inhibitory Activity Assay

The COX-2 inhibitory activity assay of sample was conducted according to a previously reported method [16]. Compounds were evaluated for potency and selectivity of inhibition in vitro using Cayman’s COX Fluorescent Inhibitor Screening Assay Kit (Cayman, #700100). Human recombinant COX-2 enzyme was pre-incubated with inhibitors for 15 min at normal temperature, followed by the addition of heme and fluorometric substrate and incubation for another 15 min at normal temperature. The reaction was started by the addition of arachidonic acid and allowed to proceed for 2 min. The fluorescence was measured at 530 nm excitation wavelength and 595 nm emission wavelength using a micro plate reader (Envision, PerkinElmer). The final concentrations of each compound in the wells were confined as 30, 10, 3.3, 1.1, 0.37, 0.12, 0.04, and 0.013 μM. The DMSO and Celecoxib were used for a blank and positive control. Each assay was carried out in triplicates. IC_50_, was measured by non-liner regression.

## 4. Conclusions

In conclusion, this study has revealed three new and three known fungal metabolites from the EtOAc extract of the marine sponge-derived fungal strain, *Alternaria* sp. SCSIO S02F49. Compounds **1** and **2** possess an unusual altenusin-thiazole-fused skeleton, and **3** is the first benzothiazole derivative isolated from fungi. The thiazole core of **1**–**3** may originate from NAD (nicotinamide adenine dinucleotide) and glycine [17]. Compounds **4** and **5** showed higher DPPH free-radical-scavenging activities than BHT. Additionally, **5** exhibited moderate COX-2 inhibitory activity with an IC_50_ value of 9.5 ± 0.08 μM. Our work enriches the structure diversity of marine fungal natural products.

## Figures and Tables

**Table 1:** ^1^H and ^13^C NMR data for **1**, **2**, and **4** (500/125 MHz, in DMSO, δ ppm, *J* in Hz).

No.	1			2			4	
	δ_H_ (CD_3_OD)	δ_H_ (*J* in Hz)	δ_C_	δ_H_ (CD_3_OD)	δ_H_ (*J* in Hz)	δ_C_	δ_H_ (*J* in Hz)	δ_C_
1			143.2, C			144.0, C		145.0, C
2			106.8, C			108.2, C		108.8, C
3			163.4, C			162.1, C		161.6, C
4	6.47, d (2.4)	6.57, d (2.4)	100.7, CH	6.42, d (2.0)	6.50, d (2.0)	100.2, CH	6.44, d (2.7)	99.6, CH
5			163.3, C			162.6, C		163.0, C
6	6.17, d (2.4)	6.24, d (2.4)	109.2, CH	6.14, d (2.0)	6.19, d (2.0)	108.8, CH	6.10, d (2.7)	108.9, CH
7			171.6, C			171.5, C		171.6, C
8	3.80, s	3.79, s	55.6, CH_3_	3.79, s	3.78, s	55.5, CH_3_	3.76, s	55.3, CH_3_
1′			126.0, C			139.0, C		132.4, C
2′			132.9, C			118.1, C		124.9, C
3′	6.77, s	6.78, s	113.3, CH			135.8, C	6.54, s	116.6, CH
4′			150.1, C			141.4, C		143.9, C
5′			140.3, C			148.6, C		142.1, C
6′			136.1, C	6.70, s	6.64, s	112.0, CH	6.42, s	115.9, CH
7′	2.13, s	2.07, s	19.6, CH_3_	2.19, s	2.13, s	18.7, CH_3_	1.86, s	18.8, CH_3_
8′	8.83, s	9.02, s	152.0, CH	8.97, s	9.20, s	152.5, CH		
								
3-OH								
4′-OH		11.96, br.s					8.70, s	
5′-OH					10.04, s		8.65, s	
COOH		10.11, br.s					11.53, br.s	

**Table 2:** ^1^H and ^13^C NMR data for **3** (500/125 MHz, in DMSO-*d*_6_, δ ppm, *J* in Hz).

No.	δ_H_ (*J* in Hz)	δc	No.	δ_H_ (*J* in Hz)	δc
1	4.07, s	36.8, CH_2_	7	7.34, d (1.5)	105.5, CH
2	9.05, s	151.7, CH	8		134.8, C
3		145.8, C	9		171.2, C
4		129.6, C	10	3.59, s	51.7, CH_3_
5	6.91, d (1.5)	116.8, CH	6-OH	9.86, br.s	
6		155.4, C			

**Table 3:** DPPH free radical scavenging and COX-2 inhibitory activities of **1**–**6** (IC_50_, μM).

Compounds	DPPH Free Radical Scavenging Activity	COX-2 Inhibitory Activity
**1**	>1000	>30
**2**	>1000	>30
**3**	>1000	>30
**4**	10.7 ± 0.09	>30
**5**	100.6 ± 0.025	9.5 ± 0.08
**6**	>1000	>30
BHT	170.3 ± 0.06	-
Celecoxib	-	0.008

## Supplementary Materials

The following are available online, Figure S1: ^1^H NMR (500 MHz, DMSO-*d*_6_) spectrum of **1**, Figure S2: The ^13^C NMR (125 MHz, DMSO-*d*_6_) spectrum of **1**, Figure S3: The DEPT-135 (125 MHz, DMSO-*d*_6_) spectrum of **1**, Figure S4: The HSQC spectrum of **1**, Figure S5: The HMBC spectrum of **1**, Figure S6: The HRESIMS spectrum of **1**, Figure S7: ^1^H NMR (500 MHz, CD_3_OD-*d*_4_) spectrum of **1**, Figure S8: 1D NOESY of **1**, Figure S9: The ^1^H NMR (500 MHz, DMSO-*d*_6_) spectrum of **2**, Figure S10: The ^13^C NMR (125 MHz, DMSO-*d*_6_) spectrum of **2**, Figure S11: The DEPT-135 (125 MHz, DMSO-*d*_6_) spectrum of **2**, Figure S12: The HSQC spectrum of **2**, Figure S13: The HMBC spectrum of **2**, Figure S14: The HRESIMS spectrum of **2**, Figure S15: ^1^H NMR (500 MHz, CD_3_OD-*d*_4_) spectrum of **2**, Figure S16: 1D NOESY of **2**, Figure S17: The ^1^H NMR (500 MHz, DMSO-*d*_6_) spectrum of **3**, Figure S18: The ^13^C NMR (125 MHz, DMSO-*d*_6_) spectrum of **3**, Figure S19: The DEPT-135 (125 MHz, DMSO-*d*_6_) spectrum of 3, Figure S20: The HSQC spectrum of **3**, Figure S21: The HMBC spectrum of **3**, Figure S22: The HRESIMS spectrum of **3**, Figure S23: The ^1^H NMR (500 MHz, DMSO-*d*_6_) spectrum of 4, Figure S24: The ^13^C NMR (125 MHz, DMSO-*d*_6_) spectrum of **4**, Figure S25: The DEPT-135 (125 MHz, DMSO-*d*_6_) spectrum of **4**, Figure S26: The ^1^H NMR (500 MHz, DMSO-*d*_6_) spectrum of **5**, Figure S27: The ^13^C NMR (125 MHz, DMSO-*d*_6_) spectrum of **5**, Figure S28: The DEPT-135 (125 MHz, DMSO-*d*_6_) spectrum of **5**, Figure S29: The ^1^H NMR (500 MHz, DMSO-*d*_6_) spectrum of **6**, Figure S30: The ^13^C NMR (125 MHz, DMSO-*d*_6_) spectrum of **6**, Figure S31: The DEPT-135 (125 MHz, DMSO-*d*_6_) spectrum of **6**, Table S1: Crystal date and structure refinement of **3**. Table S2: ^1^H and ^13^C NMR Data for **5** and **6**.
