# Supplementary material for: Two New Altenusin/Thiazole Hybrids and a New Benzothiazole Derivative from the Marine Sponge-Derived Fungus Alternaria sp. SCSIOS02F49
Source: Molecules. 2018 Nov 1;23(11):2844. doi: 10.3390/molecules23112844 (PMC6278658; doi:10.3390/molecules23112844)
Supplement: Supplementary file 1 [file molecules-23-02844-s001.pdf]

Supplementary data

Altenusinoides A-B, Two New Altenusin/Thiazole Hybrids from the Marine Sponge-Derived Fungus *Alternaria* sp. SCSIO S02F49

Yaping Chen [1], Ruyan Chen [2], Jinhuai Xu [2], Yongqi Tian [2,*], Jiangping Xu [1,*] and Yonghong Liu [3,*]

[1] School of Pharmaceutical Sciences, Southern Medical University, Guangzhou 510515, China; E-mails: zlchenyaping@126.com (Y.C.).

[2] College of Biological science and technology, Fuzhou university, Fuzhou 350116, China; E-mails: 13107668079@163.com (R.C.); xujinhuai@yahoo.com (J.X.).

[3] CAS Key Laboratory of Tropical Marine Bio-resources and Ecology/Guangdong Key Laboratory of Marine Materia Medica/ RNAM Center for Marine Microbiology, South China Sea Institute of Oceanology, Chinese Academy of Sciences, Guangzhou 510301, China.

* Correspondence: tian.yongqi@163.com (Y.T.); jpx@smu.edu.cn (J.X.); yonghongliu@scsio.ac.cn (Y.L.); Tel./Fax: +86-020-8902-3244 (Y.L.)

Molecules **2018**, *23*, 2844; doi:10.3390/molecules23112844

Contents	Pages
**Table S1.** Crystal date and structure refinement of **3**	3
**Table S2.** ^{1}H and ^{13}C-NMR Data for **5** and **6**	4
**Figure S1**. ^{1}H NMR (500 MHz, DMSO-*d6*) spectrum of **1**	5
**Figure S2**. The ^{13}C NMR (125 MHz, DMSO-*d6*) spectrum of **1**	5
**Figure S3**. The DEPT-135 (125 MHz, DMSO-*d6*) spectrum of **1**	6
**Figure S4**. The HSQC spectrum of **1**	6
**Figure S5**. The HMBC spectrum of **1**	7
**Figure S6**. The HRESIMS spectrum of **1**	7
**Figure S7**.^{1}H NMR (500 MHz, CD_3OD-*d4*) spectrum of **1**	8
**Figure S8**. 1D NOESY of **1**	8
**Figure S9**. The ^{1}H NMR (500 MHz, DMSO-*d6*) spectrum of **2**	9
**Figure S10**. The ^{13}C NMR (125 MHz, DMSO-*d6*) spectrum of **2**	9
**Figure S11**. The DEPT-135 (125 MHz, DMSO-*d6*) spectrum of **2**	10
**Figure S12**. The HSQC spectrum of **2**	10
**Figure S13**. The HMBC spectrum of **2**	11
**Figure S14**. The HRESIMS spectrum of **2**	11
**Figure S15**.^{1}H NMR (500 MHz, CD_3OD-*d4*) spectrum of **2**	12
**Figure S16**. 1D NOESY of **2**	12
**Figure S17**. The ^{1}H NMR (500 MHz, DMSO-*d6*) spectrum of **3**	13
**Figure S18**. The ^{13}C NMR (125 MHz, DMSO-*d6*) spectrum of **3**	13
**Figure S19**. The DEPT-135 (125 MHz, DMSO-*d6*) spectrum of **3**	14
**Figure S20**. The HSQC spectrum of **3**	14
**Figure S21**. The HMBC spectrum of **3**	15
**Figure S22**. The HRESIMS spectrum of **3**	15
**Figure S23**. The ^{1}H NMR (500 MHz, DMSO-*d6*) spectrum of **4**	16
**Figure S24** The ^{13}C NMR (125 MHz, DMSO-*d6*) spectrum of **4**	16
**Figure S25**. The DEPT-135 (125 MHz, DMSO-*d6*) spectrum of **4**	17
**Figure S26**. The ^{1}H NMR (500 MHz, DMSO-*d6*) spectrum of **5**	17
**Figure S27**. The ^{13}C NMR (125 MHz, DMSO-*d6*) spectrum of **5**	18
**Figure S28**. The DEPT-135 (125 MHz, DMSO-*d6*) spectrum of **5**	18
**Figure S29**. The ^{1}H NMR (500 MHz, DMSO-*d6*) spectrum of **6**	19
**Figure S30**. The ^{13}C NMR (125 MHz, DMSO-*d6*) spectrum of **6**	19
**Figure S31**. The DEPT-135 (125 MHz, DMSO-*d6*) spectrum of **6**	20
**Figure S32**. Phylogenetic trees of Alternaria sp. SCSIO S02F49	20

Table S1. Crystal date and structure refinement of **3**

**Identification code**	**Compound 3**	
Empirical formula	$C_{10}H_9 NO_3S$	
Formula weight	223.24	
Temperature	150(2) K	
Wavelength	1.54184 Å	
Crystal system	Triclinic	
Space group	*P*-1	
Unit cell dimensions	a = 7.4592(18) Å	α= 79.413(13)°.
	b = 8.3759(18) Å	β= 80.323(15)°.
	c = 8.7755(10) Å	γ = 65.02(2)°.
Volume	486.00(18) $Å^3$	
Z	2	
Density (calculated)	1.526 Mg/m^3	
Absorption coefficient	2.864 mm^{-1}	
F(000)	232	
Crystal size	0.370 x 0.240 x 0.170 mm^3	
Theta range for data collection	5.154 to 66.778°.	
Index ranges	-6<=h<=8, -8<=k<=9, -10<=l<=10	
Reflections collected	2995	
Independent reflections	1684 [R(int) = 0.0369]	
Completeness to theta = 66.778°	98.0 %	
Absorption correction	Semi-empirical from equivalents	
Max. and min. transmission	1.00000 and 0.51489	
Refinement method	Full-matrix least-squares on F^2	
Data / restraints / parameters	1684 / 0 / 141	
Goodness-of-fit on F^2	1.069	
Final R indices [I>2sigma(I)]	R1 = 0.0441, wR2 = 0.1138	
R indices (all data)	R1 = 0.0519, wR2 = 0.1235	
Extinction coefficient	n/a	
Largest diff. peak and hole	0.286 and -0.523 e.$Å^{-3}$	

Table S2. ^{1}H and ^{13}C-NMR Data for **5** and **6**

No.	**5**		**6**	
	δ_H mult (*J* in Hz)	δc	δ_H mult (*J* in Hz)	δc
1		144.8, C		133.9, C
2	6.21, s	109.3, CH	6.28, s	106.0, CH
3		158.5, C		160.6, C
4	6.25, s	99.7, CH	6.24, s	100.8, CH
5		160.4, C		158.7, C
6	6.20, s	106.2, CH	6.32, s	109.0, CH
7	3.69, s	55.3, CH_3	3.70, s	55.4, CH_3
1′		132.7, C		137.2, C
2′		125.3, C		169.6, C
3′	6.60, s	118.0, CH	2.95, dd (18.2 ,6.8)	41.3, CH_2
			2.40, d (18.2)	
4′		143.5, C	4.16, dd (6.6, 2.7)	71.1, CH
5′		144.5, C		206.9, C
6′	6.54, s	117.1, CH	2.11, s	18.6, CH_3
7'	2.05, s	19.8, CH_3		
3-OH	9.47, brs		9.53, brs	
4′-OH	8.83, brs			
5′-OH	8.78, brs			

Figure S1. ^{1}H **NMR (500 MHz, DMSO-*d*$_6$) spectrum of 1**

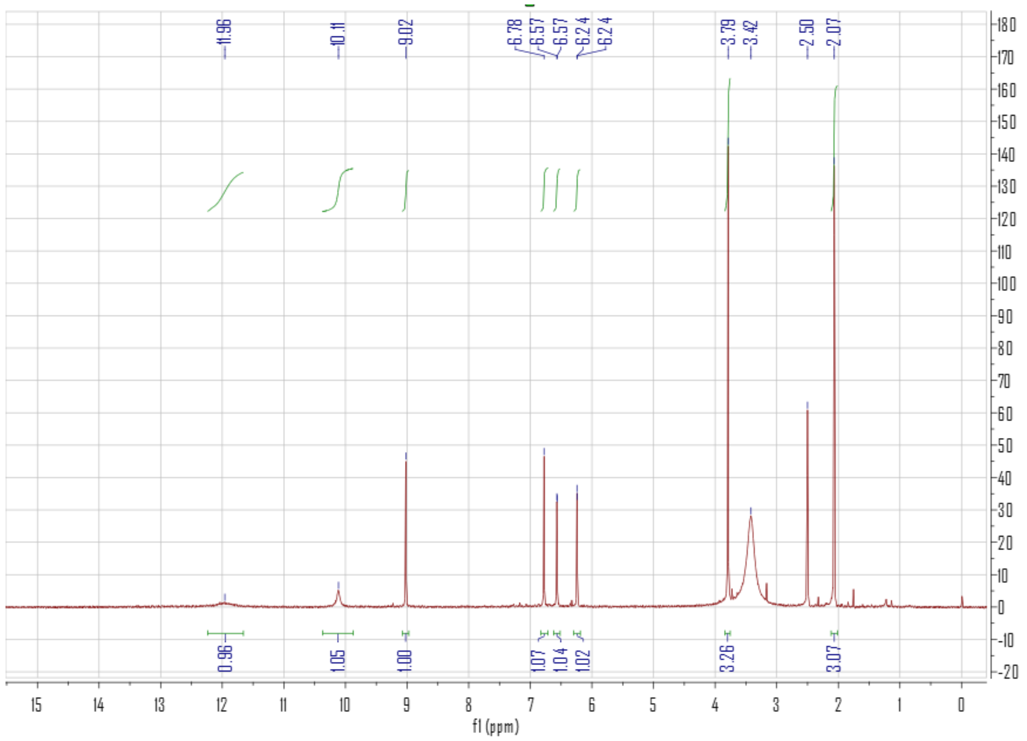

Figure S2. The ^{13}C **NMR (125 MHz, DMSO-*d6*) spectrum of 1 in DMSO**

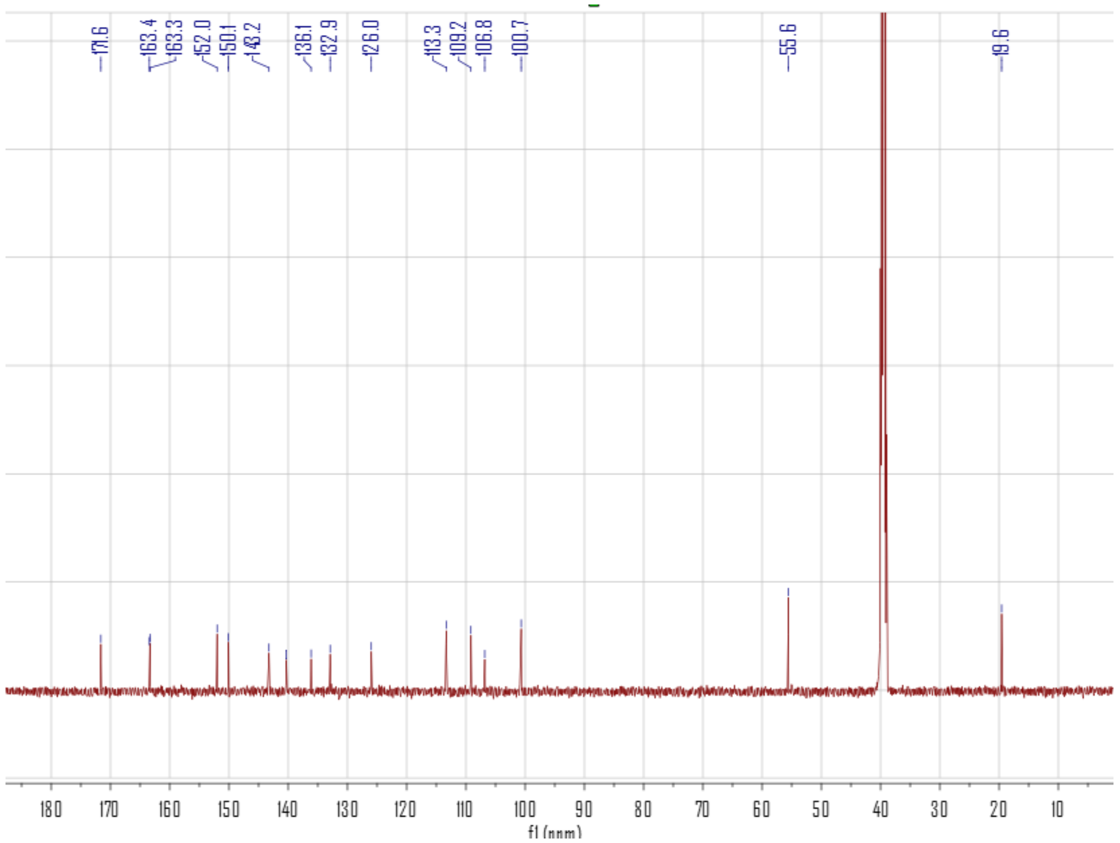

Figure S3. The DEPT-135 (125 MHz, DMSO-d_6) spectrum of 1

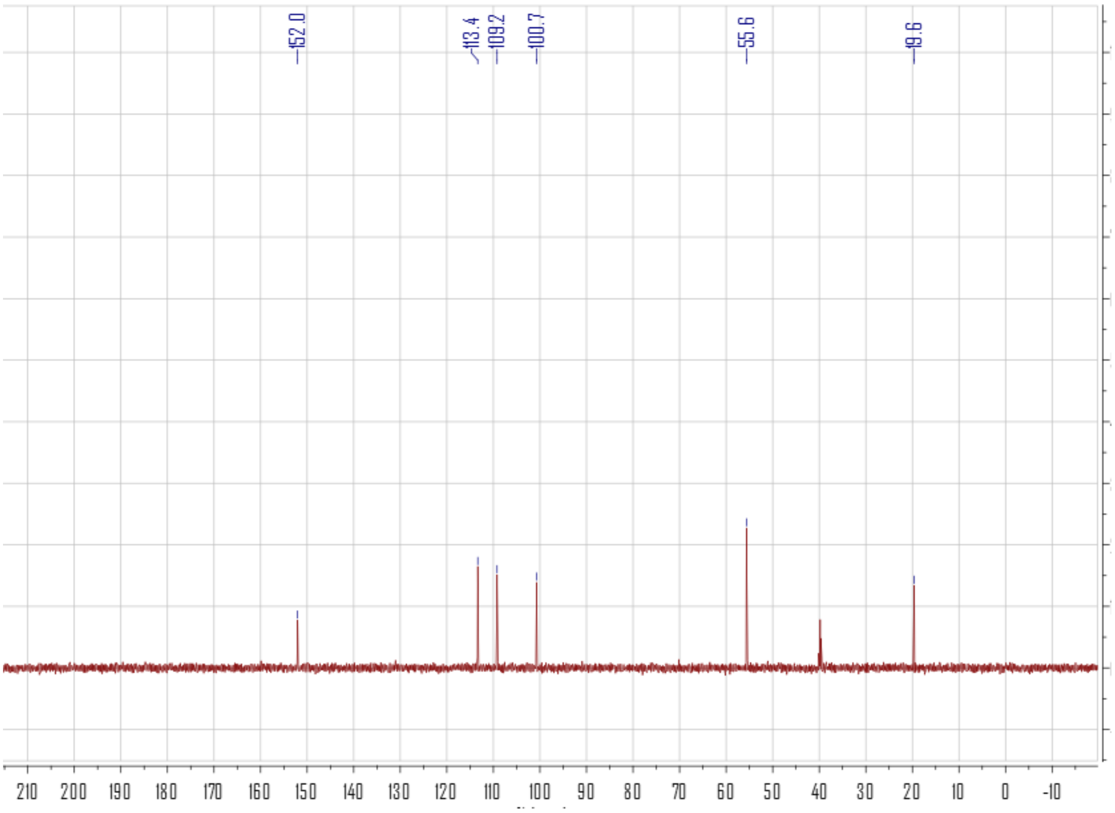

Figure S4. The HSQC spectrum of 1

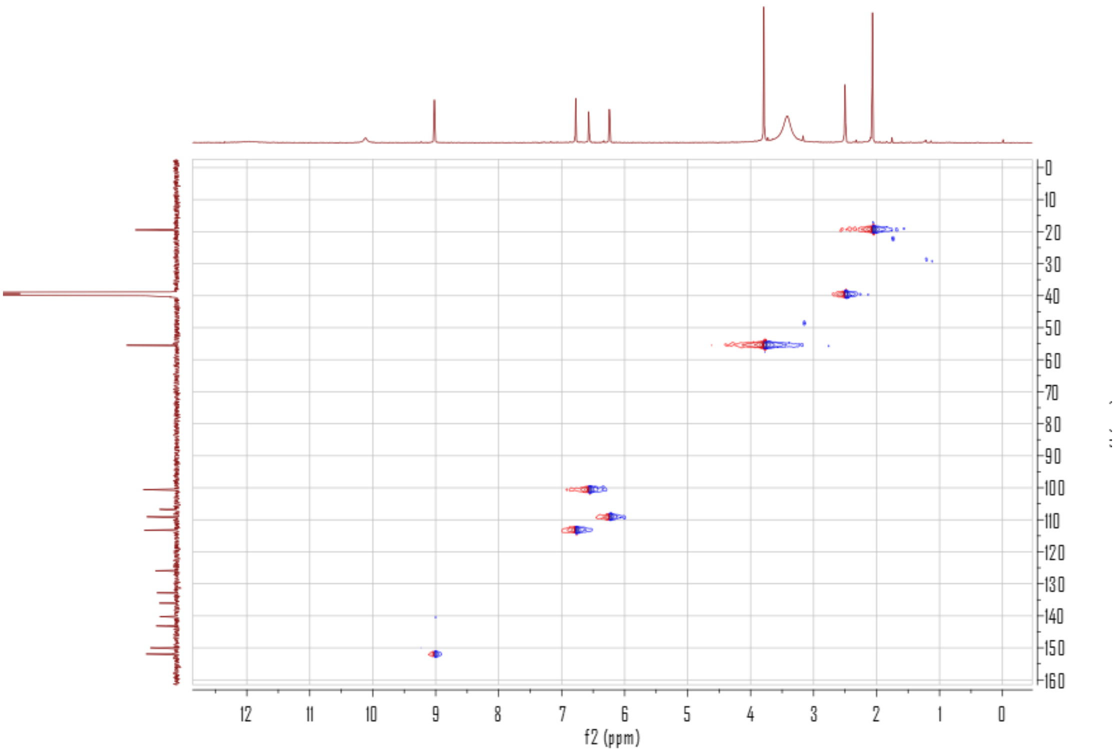

Figure S5. The HMBC spectrum of 1

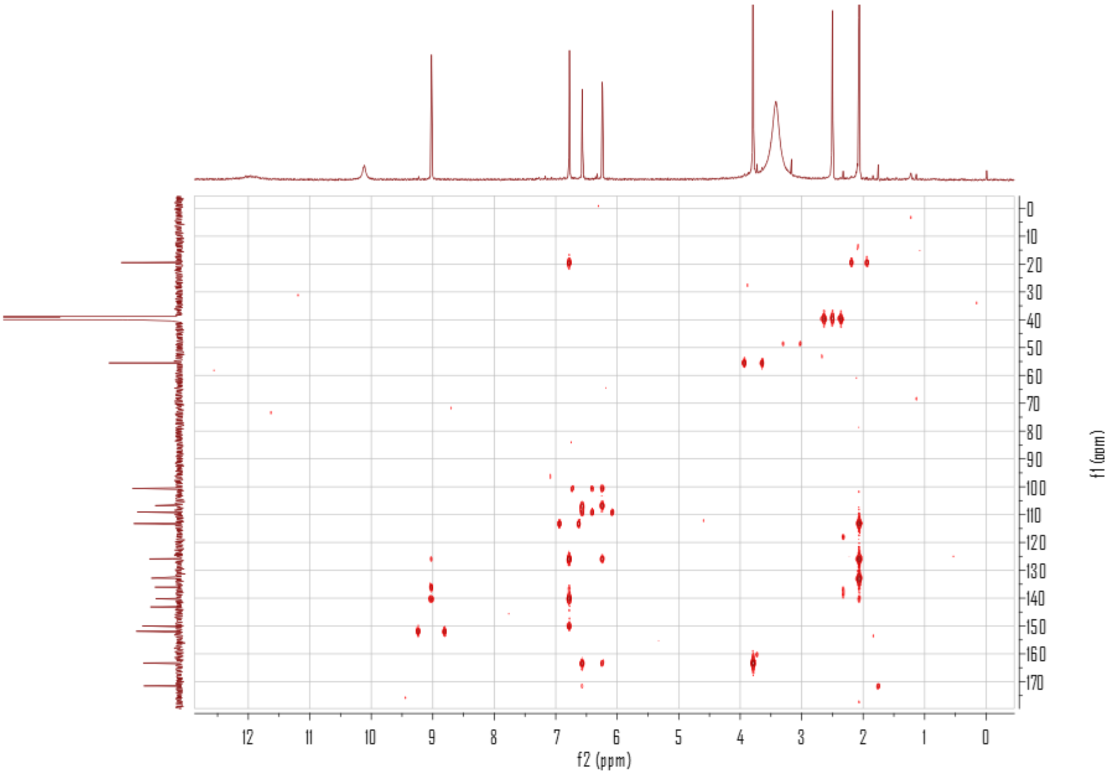

Figure S6. The HRESIMS spectrum of 1

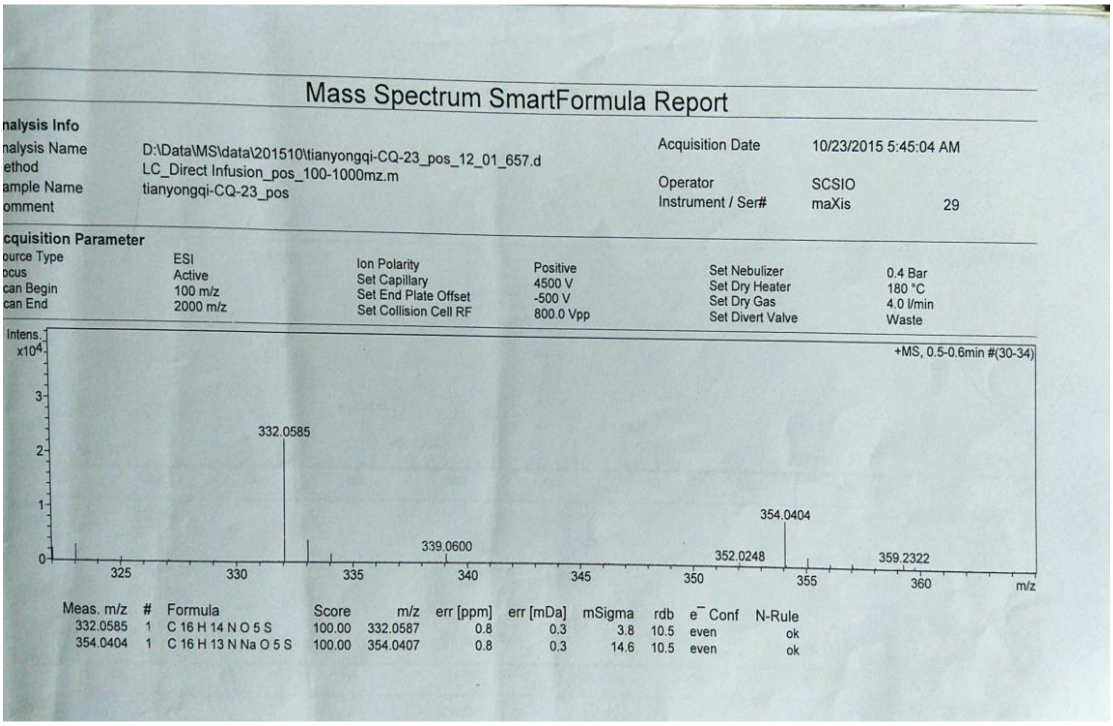

Figure S7. The ^{1}H NMR (500 MHz, CD_3OD-d_4) spectrum of 1

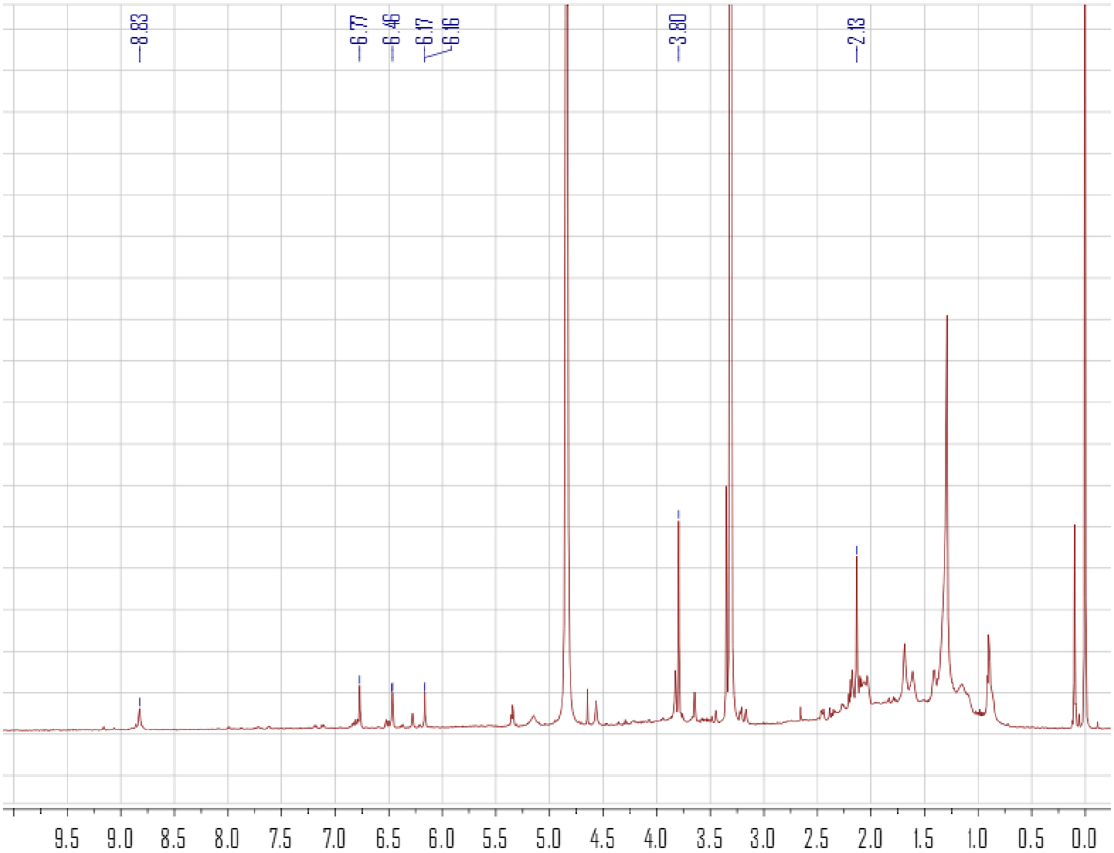

Figure S8. 1D NOESY of 1

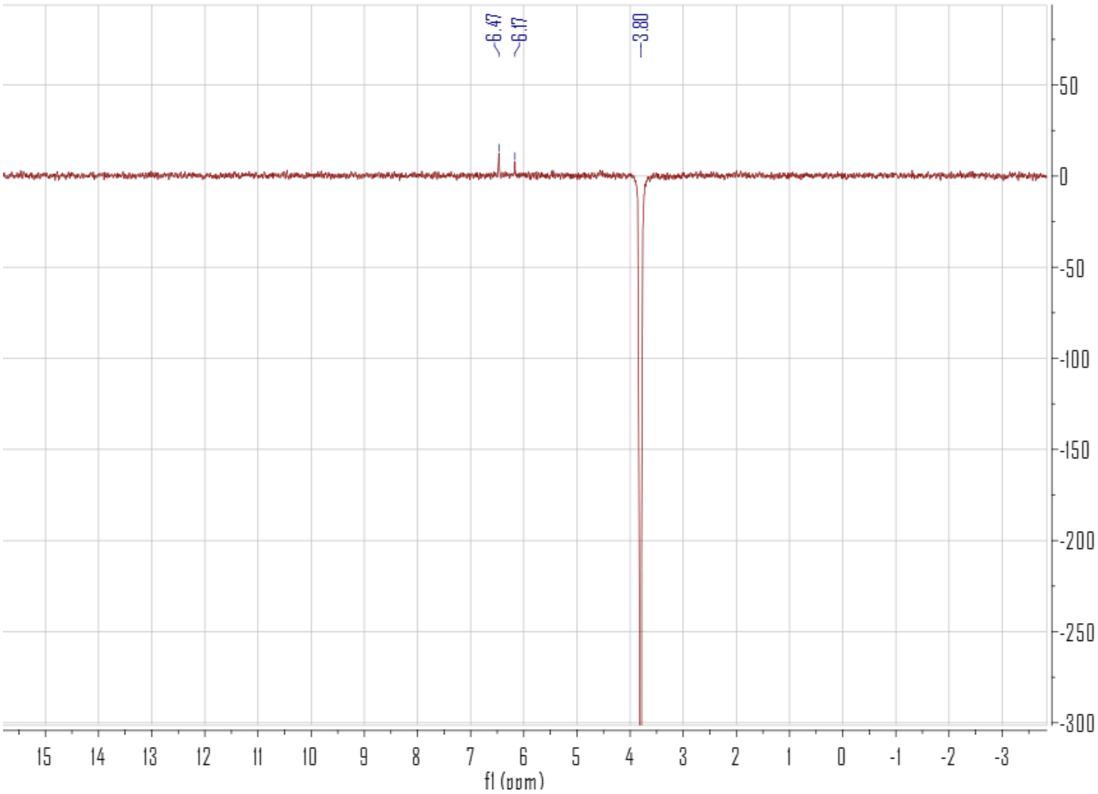

Figure S9. The ^{1}H NMR (500 MHz, DMSO-*d6*) spectrum of 2

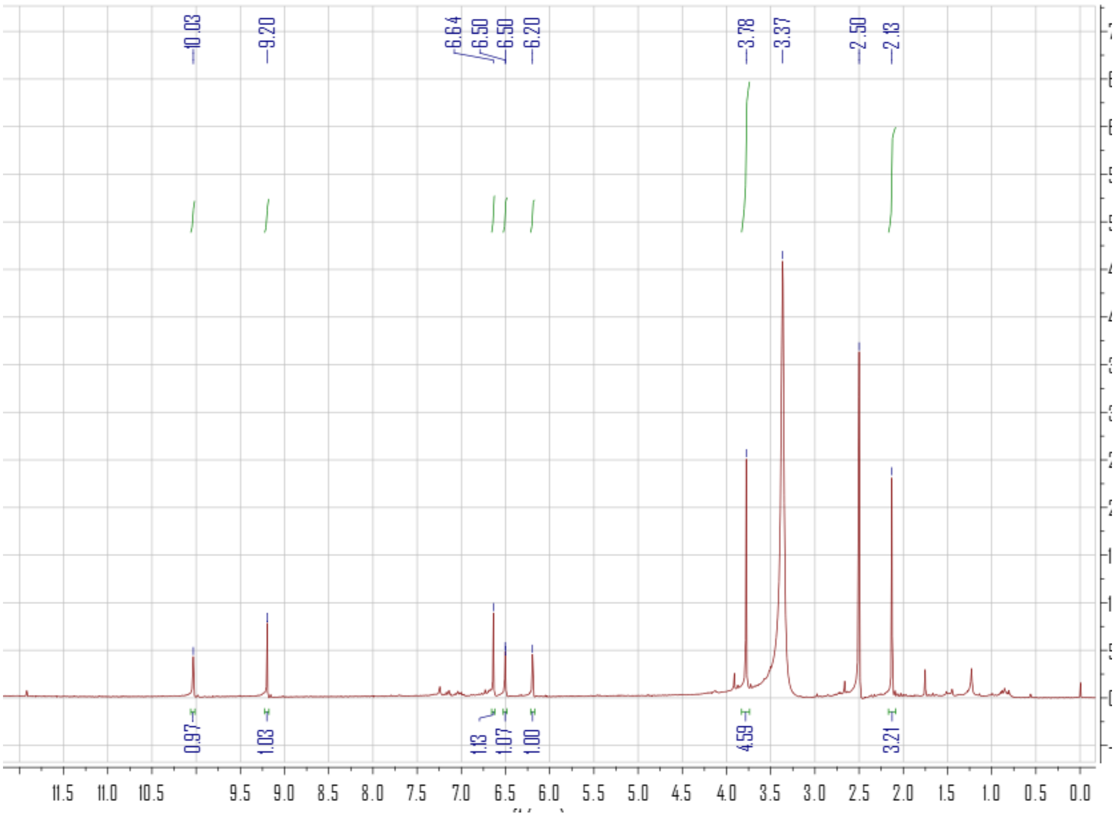

Figure S10. The ^{13}C NMR (125 MHz, DMSO-*d6*) spectrum of 2

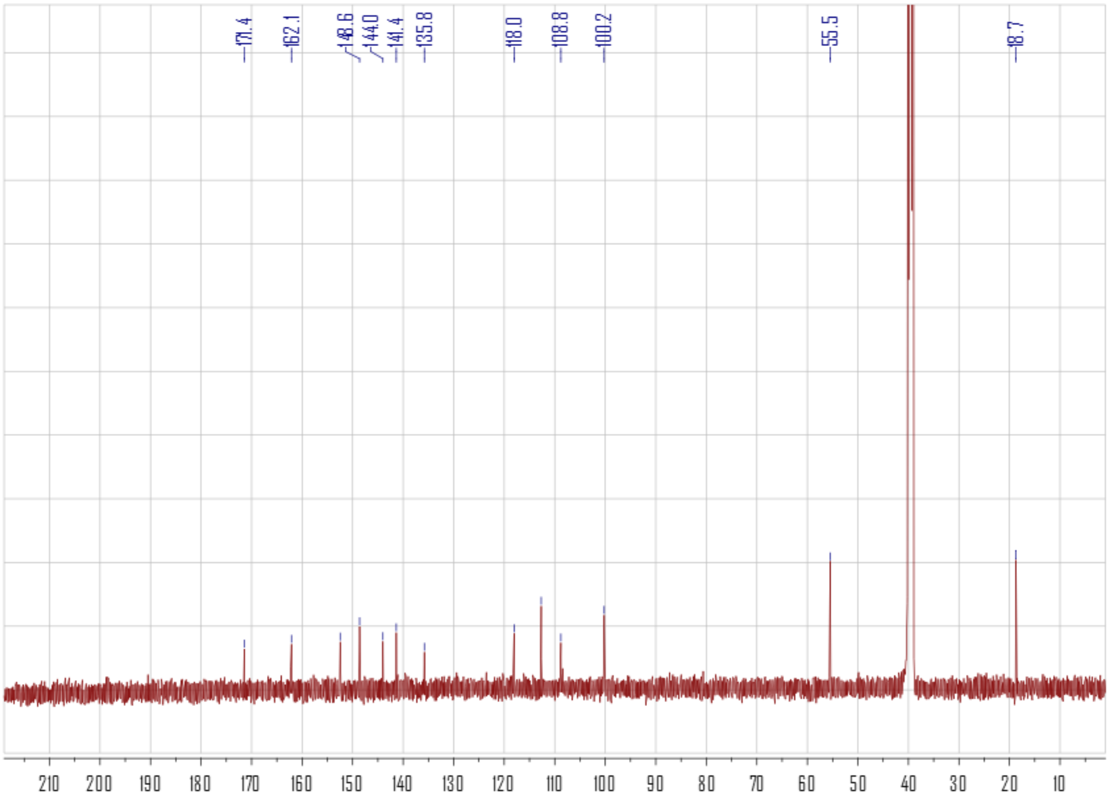

Figure S11. The DEPT-135 (125 MHz, DMSO-*d6*) spectrum of 2

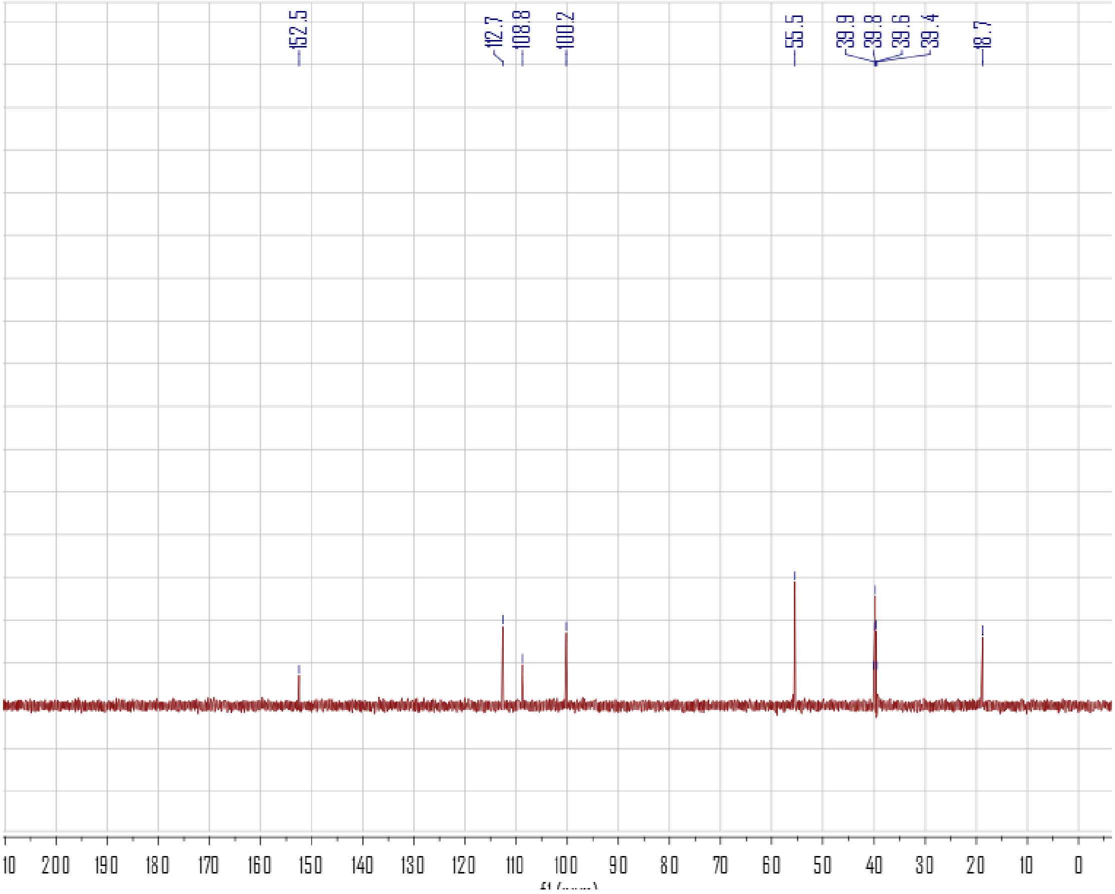

Figure S12. The HSQC spectrum of 2

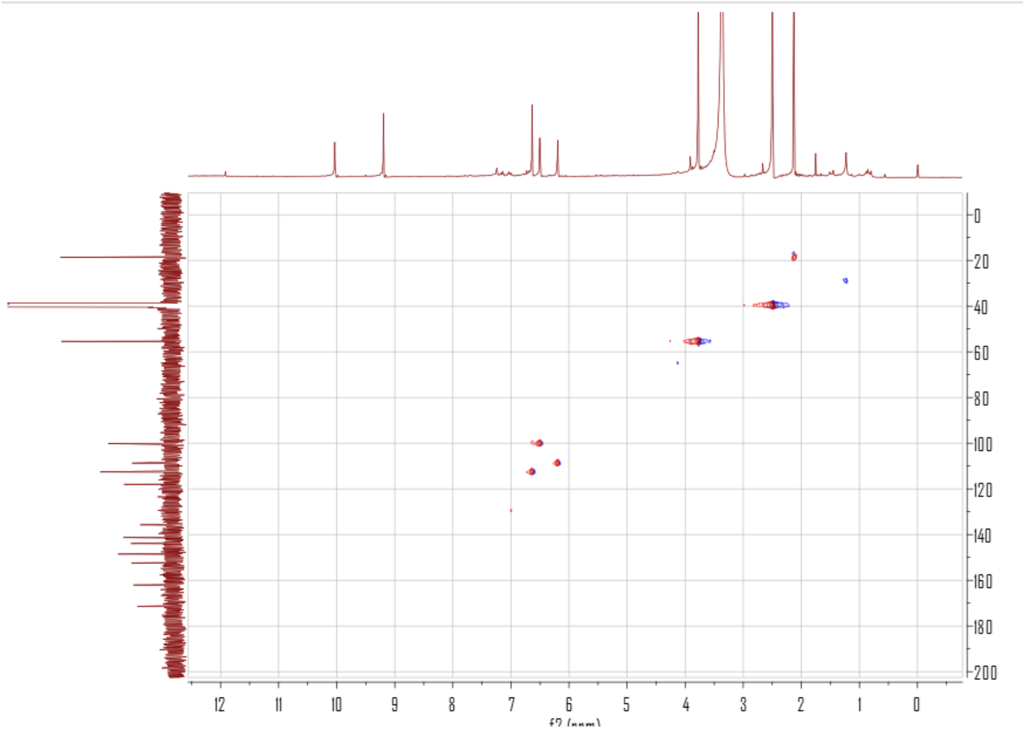

Figure S13. The HMBC spectrum of 3

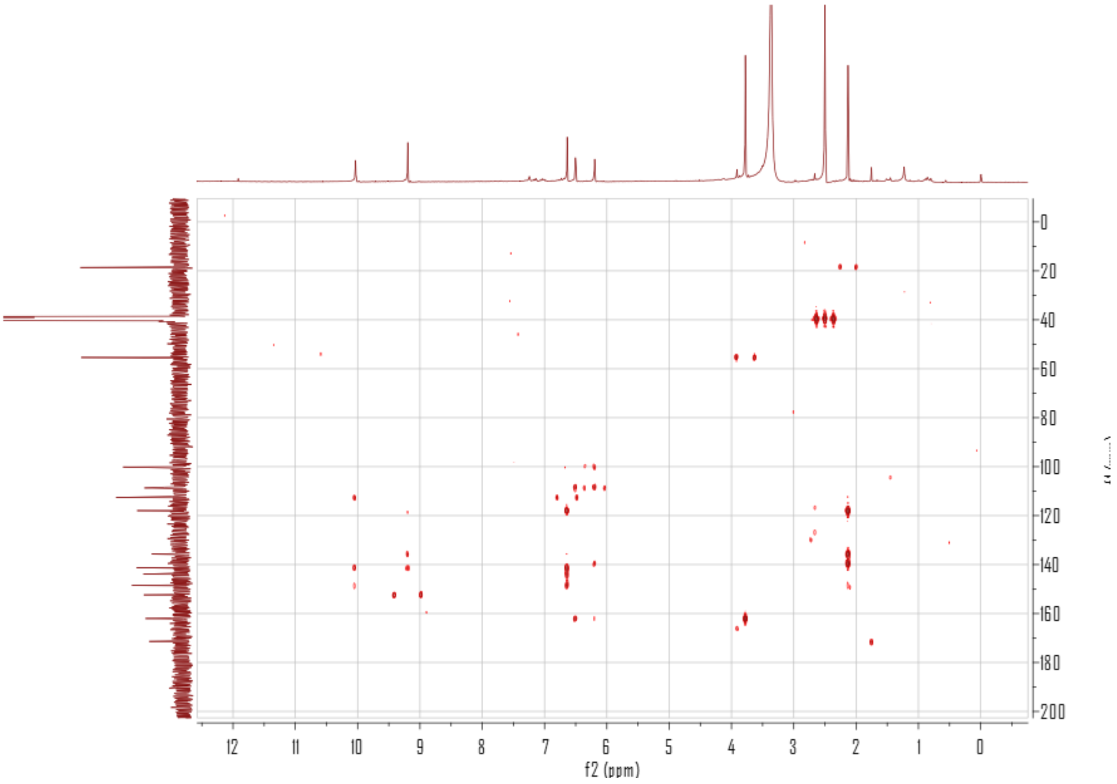

Figure S14. The HRESIMS spectrum of 3

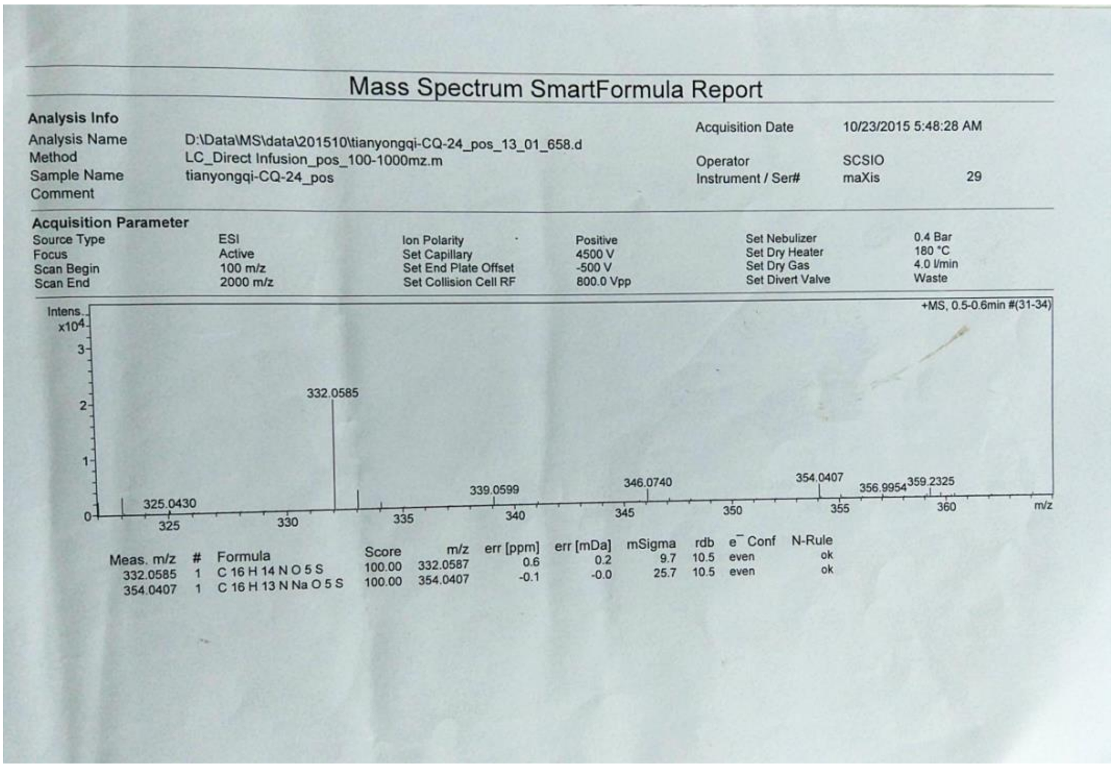

Mass Spectrum SmartFormula Report

Analysis Info

Analysis Name	D:\Data\MS\data\201510\tianyongqi-CQ-24_pos_13_01_658.d	Acquisition Date	10/23/2015 5:48:28 AM
Method	LC_Direct Infusion_pos_100-1000mz.m	Operator	SCSIO
Sample Name	tianyongqi-CQ-24_pos	Instrument / Ser#	maXis 29
Comment			

Acquisition Parameter

Source Type	ESI	Ion Polarity	Positive	Set Nebulizer	0.4 Bar
Focus	Active	Set Capillary	4500 V	Set Dry Heater	180 °C
Scan Begin	100 m/z	Set End Plate Offset	-500 V	Set Dry Gas	4.0 l/min
Scan End	2000 m/z	Set Collision Cell RF	800.0 Vpp	Set Divert Valve	Waste

Meas. m/z	#	Formula	Score	m/z	err [ppm]	err [mDa]	mSigma	rdb	e⁻ Conf	N-Rule
332.0585	1	C 16 H 14 N O 5 S	100.00	332.0587	0.6	0.2	9.7	10.5	even	ok
354.0407	1	C 16 H 13 N Na O 5 S	100.00	354.0407	-0.1	-0.0	25.7	10.5	even	ok

Figure S15. The ^{1}H NMR (500 MHz, CD_3OD-d_4) spectrum of 2

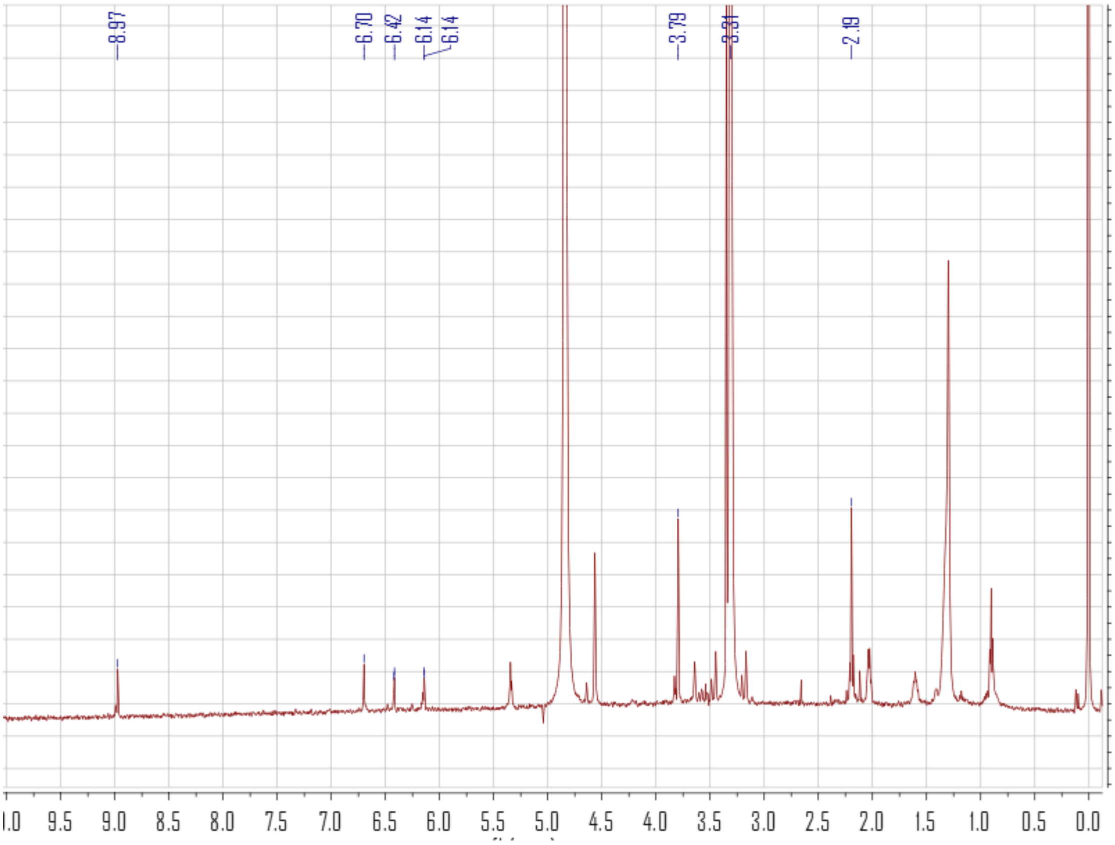

Figure S16. 1D NOESY of 2

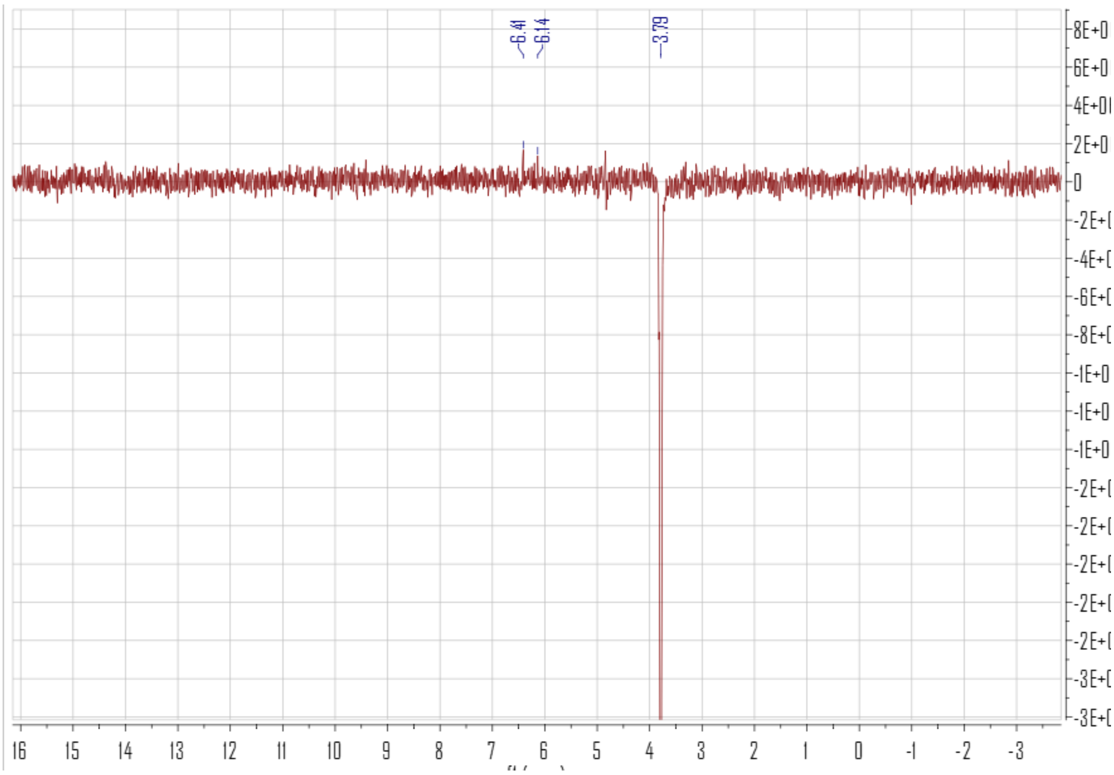

Figure S17. The ^{1}H NMR (500 MHz, DMSO-d_6) spectrum of 3

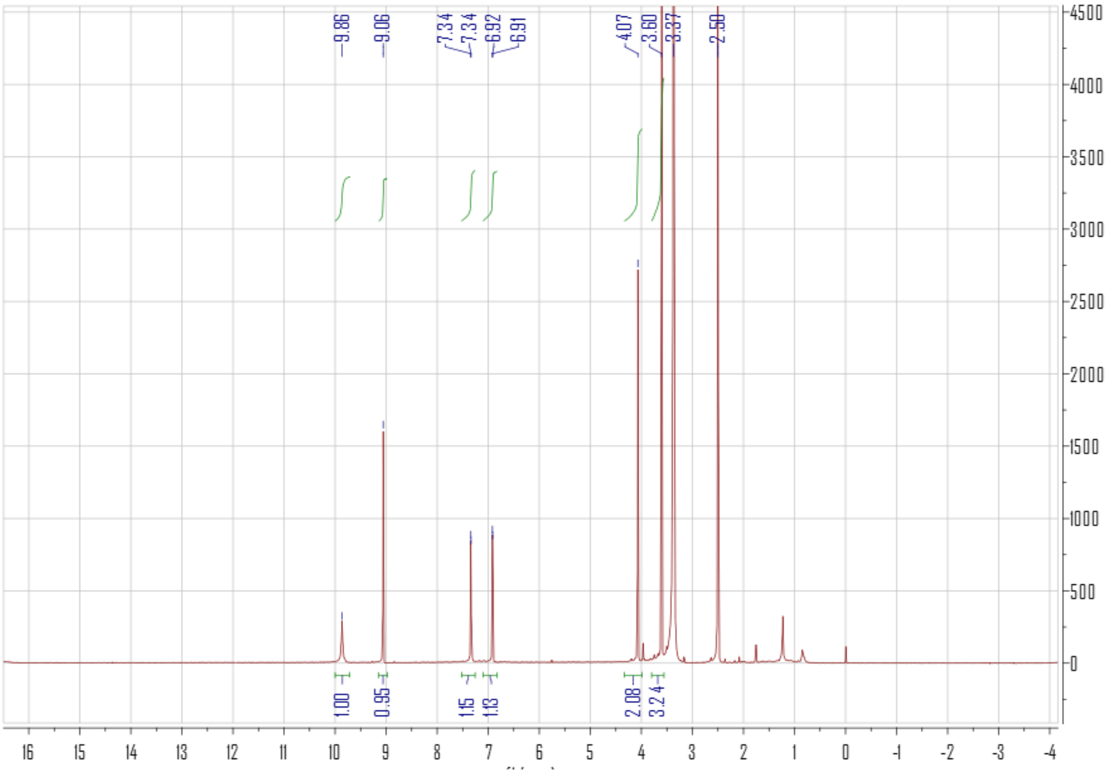

Figure S18. The ^{13}C NMR spectrum (125 MHz, DMSO-d_6) of 3

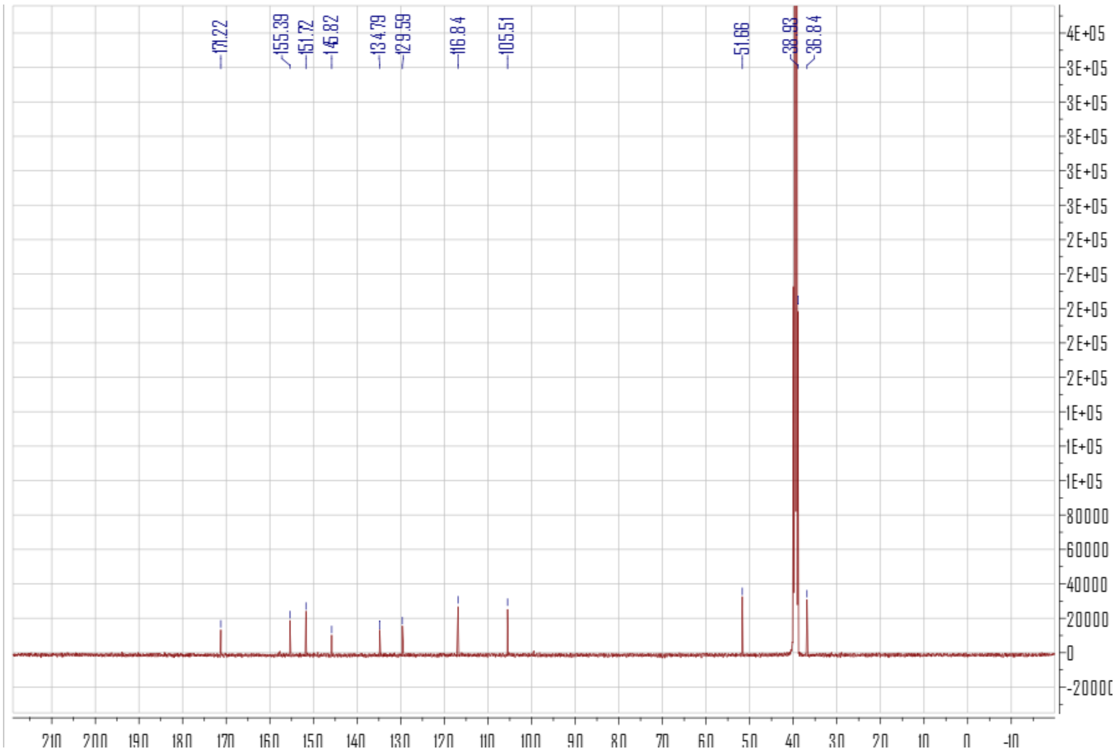

Figure S19. The DEPT-135 (125 MHz, DMSO-d_6) spectrum of 3

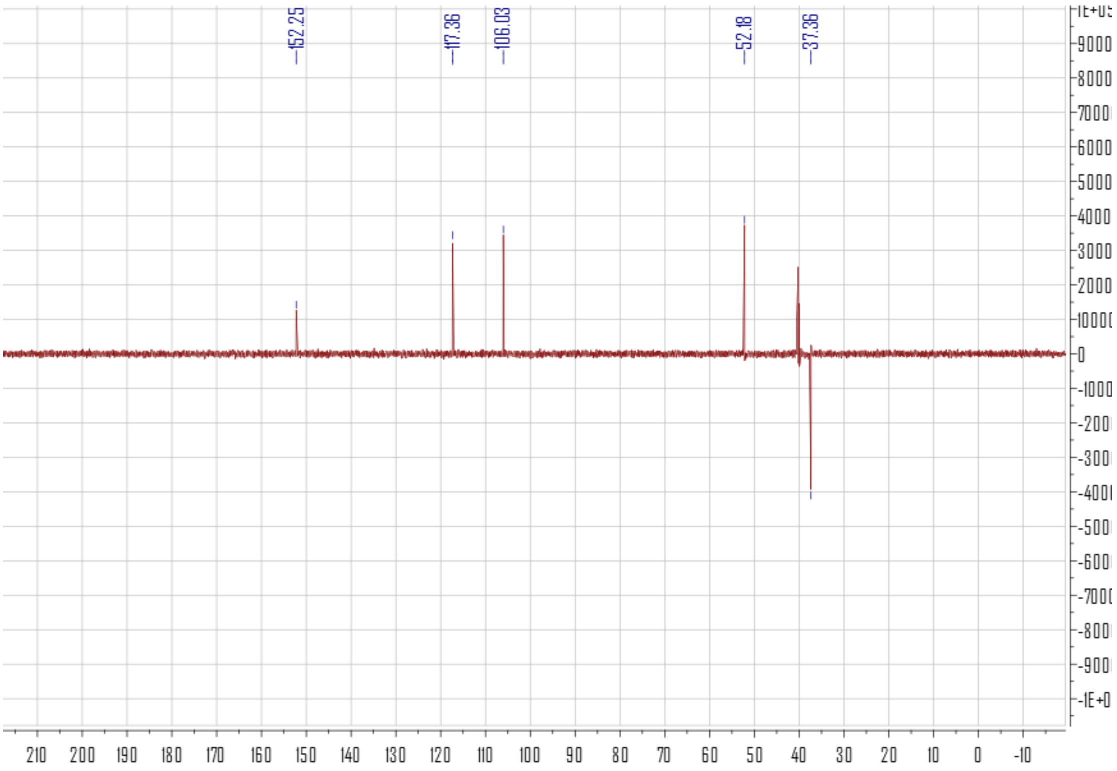

Figure S20. The HSQC spectrum of 3

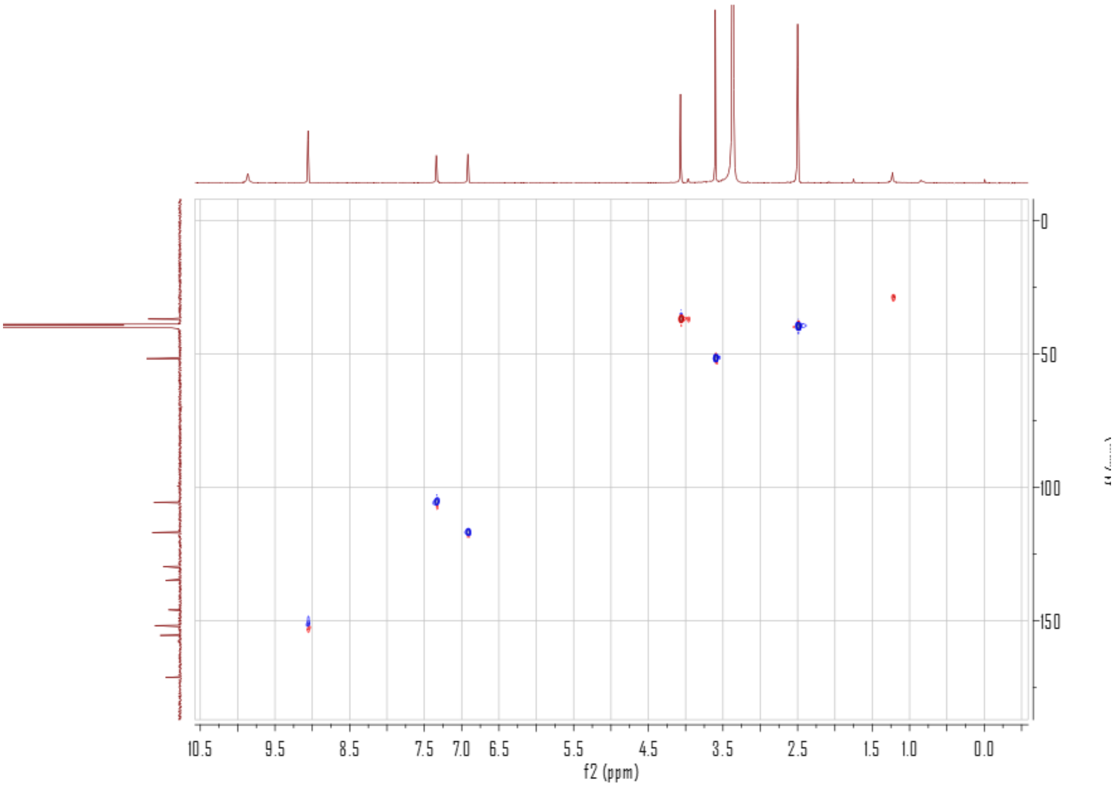

Figure S21. The HMBC spectrum of 3

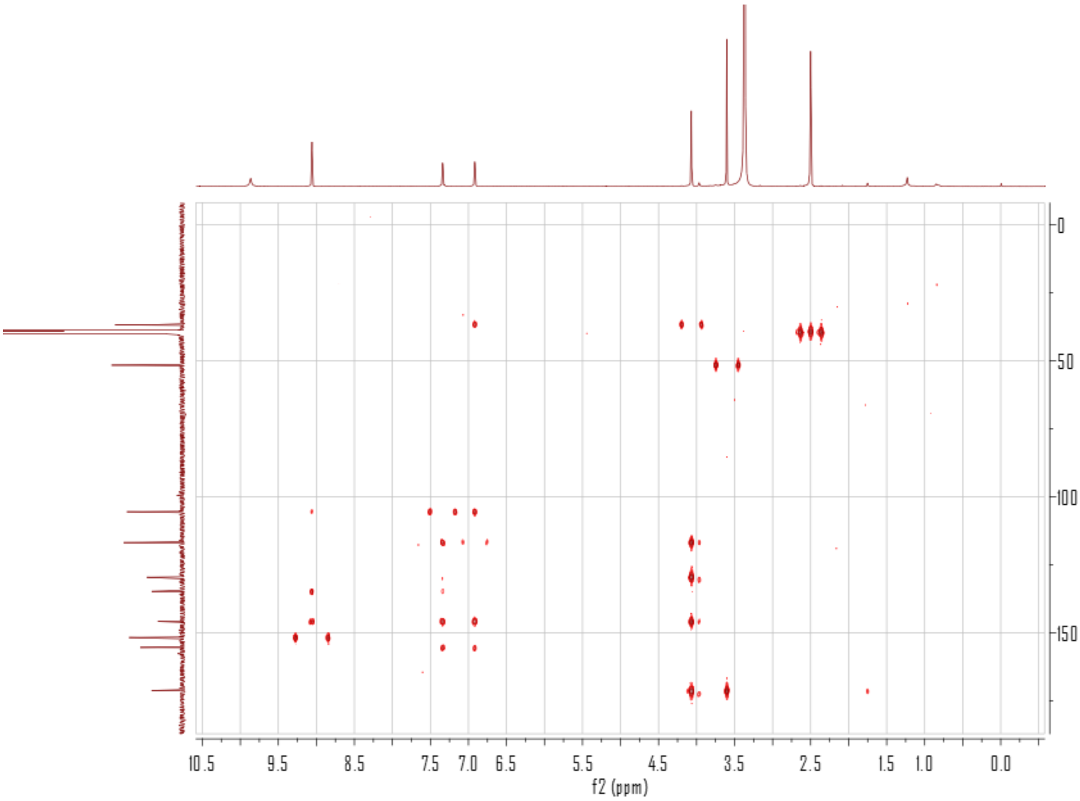

Figure S22. The HRESIMS spectrum of 3

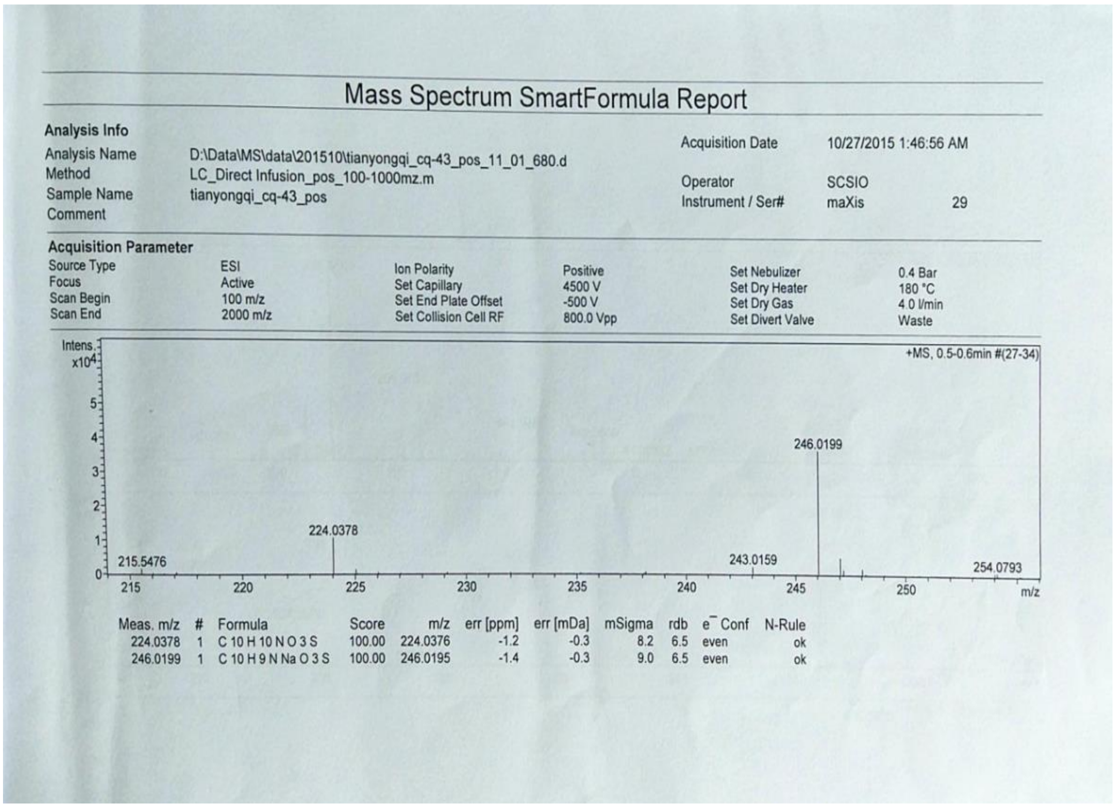

Mass Spectrum SmartFormula Report

Analysis Info

Analysis Name	D:\Data\MS\data\201510\tianyongqi_cq-43_pos_11_01_680.d	Acquisition Date	10/27/2015 1:46:56 AM
Method	LC_Direct Infusion_pos_100-1000mz.m		
Sample Name	tianyongqi_cq-43_pos	Operator	SCSIO
Comment		Instrument / Ser#	maXis 29

Acquisition Parameter

Source Type	ESI	Ion Polarity	Positive	Set Nebulizer	0.4 Bar
Focus	Active	Set Capillary	4500 V	Set Dry Heater	180 °C
Scan Begin	100 m/z	Set End Plate Offset	-500 V	Set Dry Gas	4.0 l/min
Scan End	2000 m/z	Set Collision Cell RF	800.0 Vpp	Set Divert Valve	Waste

Meas. m/z	#	Formula	Score	m/z	err [ppm]	err [mDa]	mSigma	rdb	e⁻ Conf	N-Rule
224.0378	1	C 10 H 10 N O 3 S	100.00	224.0376	-1.2	-0.3	8.2	6.5	even	ok
246.0199	1	C 10 H 9 N Na O 3 S	100.00	246.0195	-1.4	-0.3	9.0	6.5	even	ok

Figure S23. The ^{1}H NMR (500 MHz, DMSO-d_6) spectrum of 4

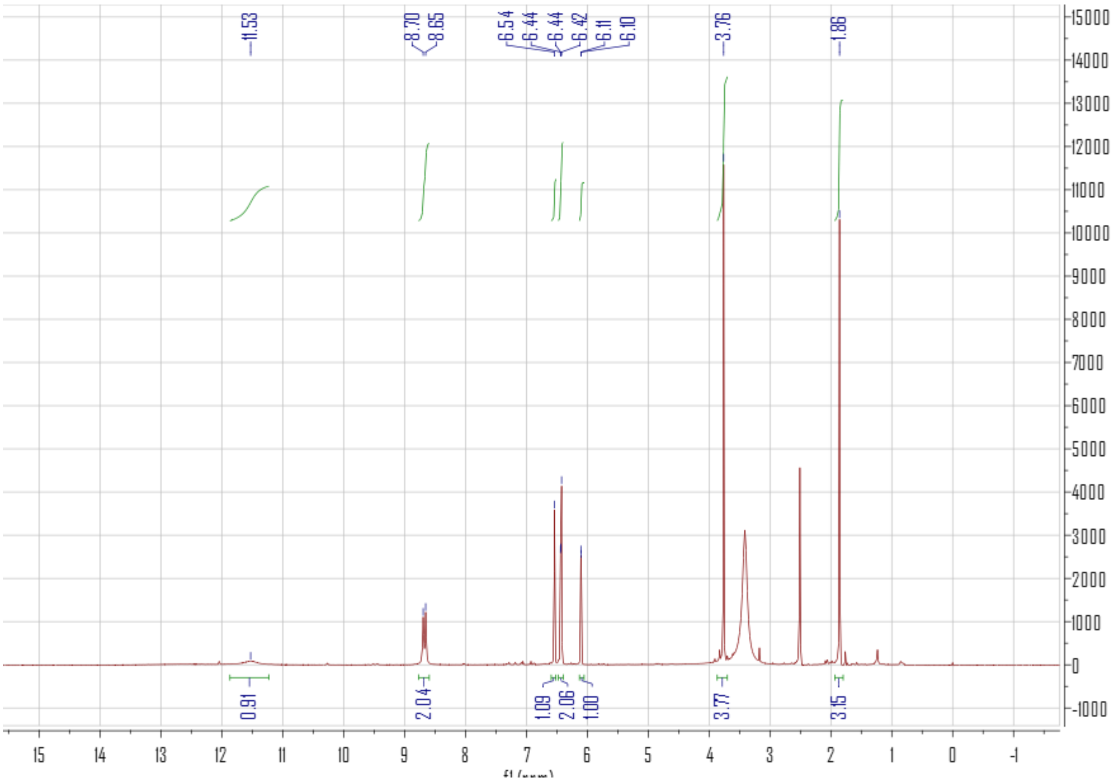

Figure S24. The ^{13}C NMR (125 MHz, DMSO-d_6) spectrum of 4

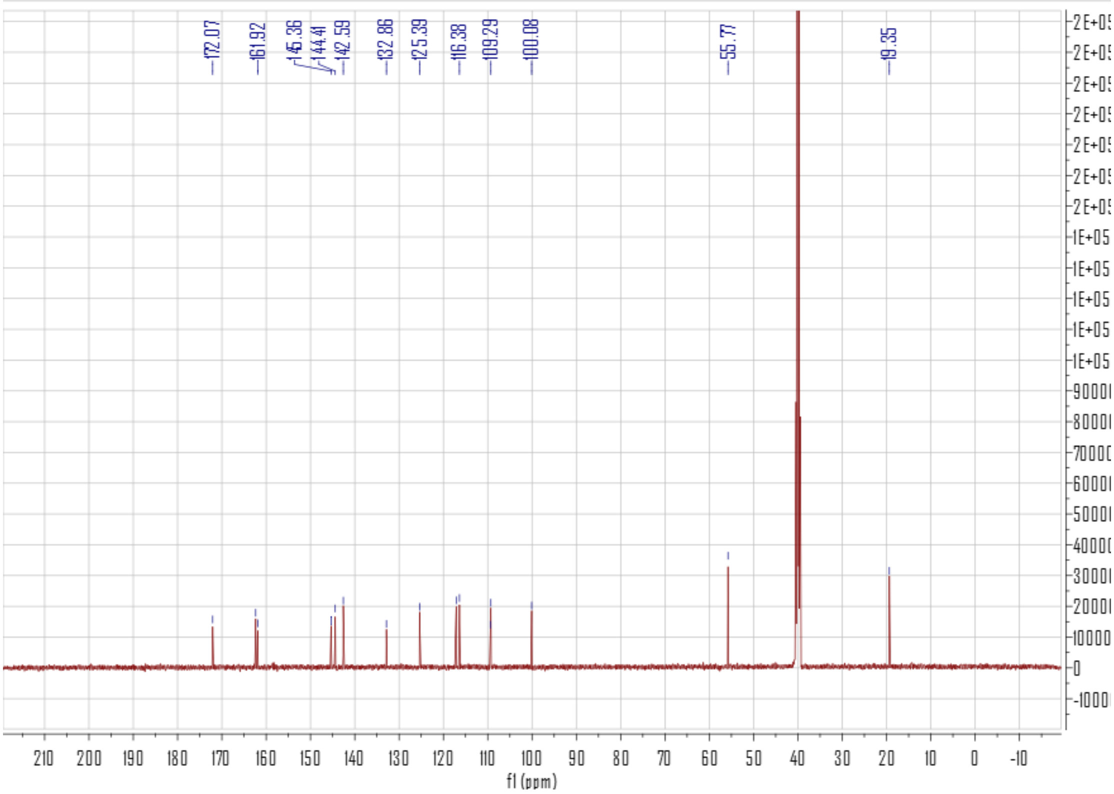

Figure S25. The DEPT (125 MHz, DMSO-d_6) spectrum of 4

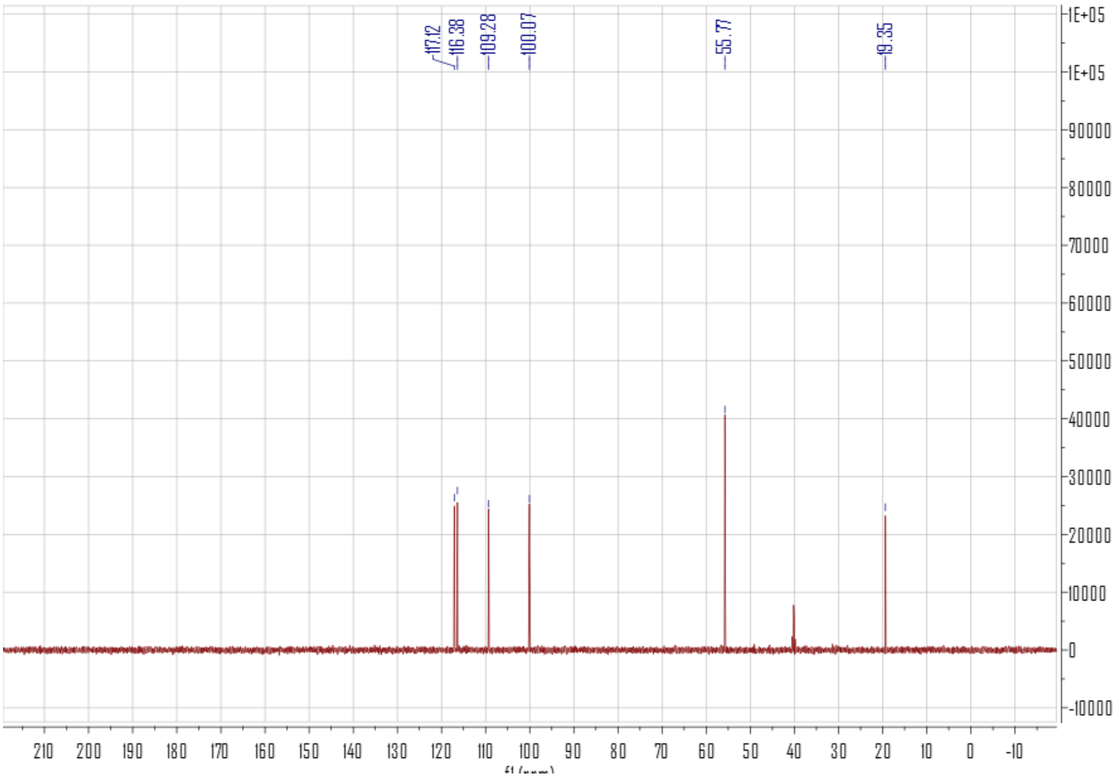

Figure S26. The ^{1}H NMR (500 MHz, DMSO-d_6) spectrum of 5

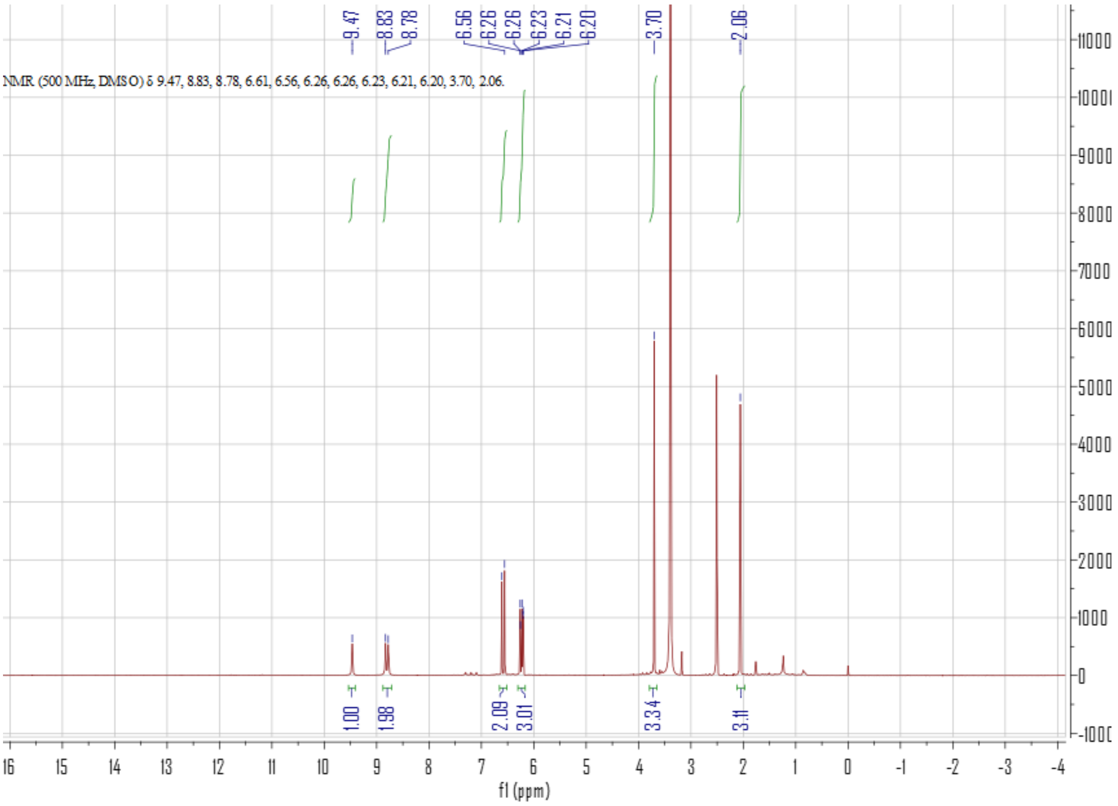

Figure S27. The ^{13}C NMR (125 MHz, DMSO-*d*$_6$) spectrum of 5

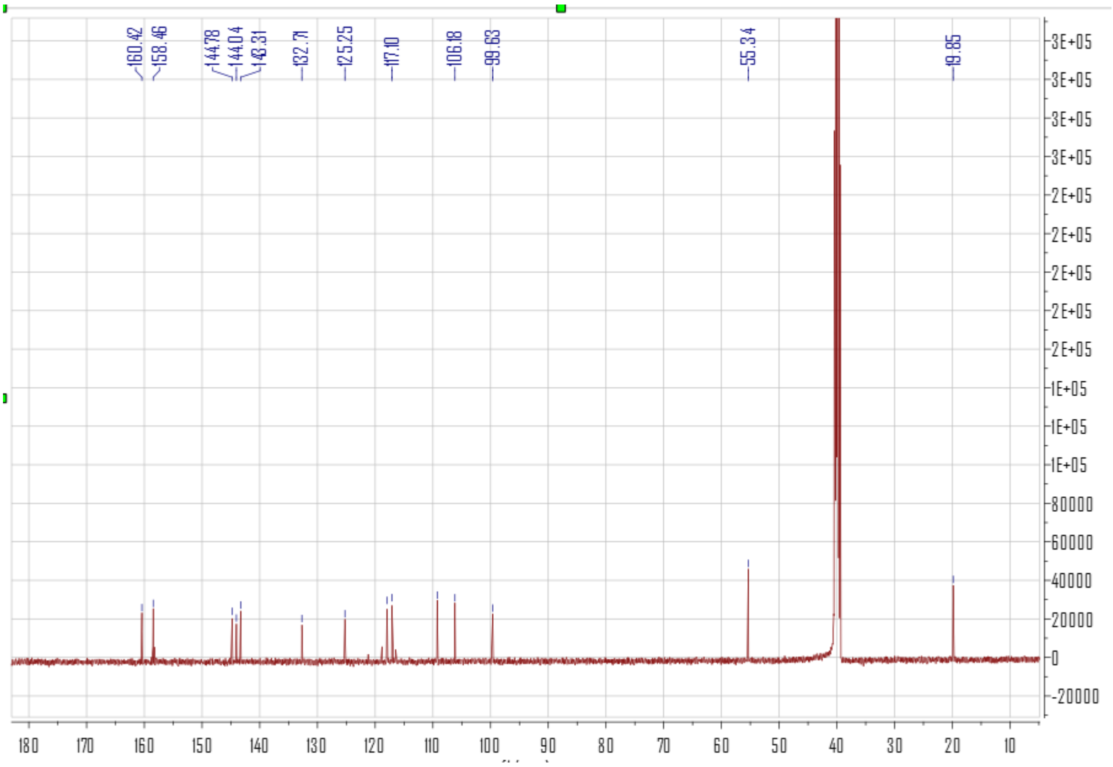

Figure S28. The DEPT-135 (125 MHz, DMSO-*d*$_6$) spectrum of 5

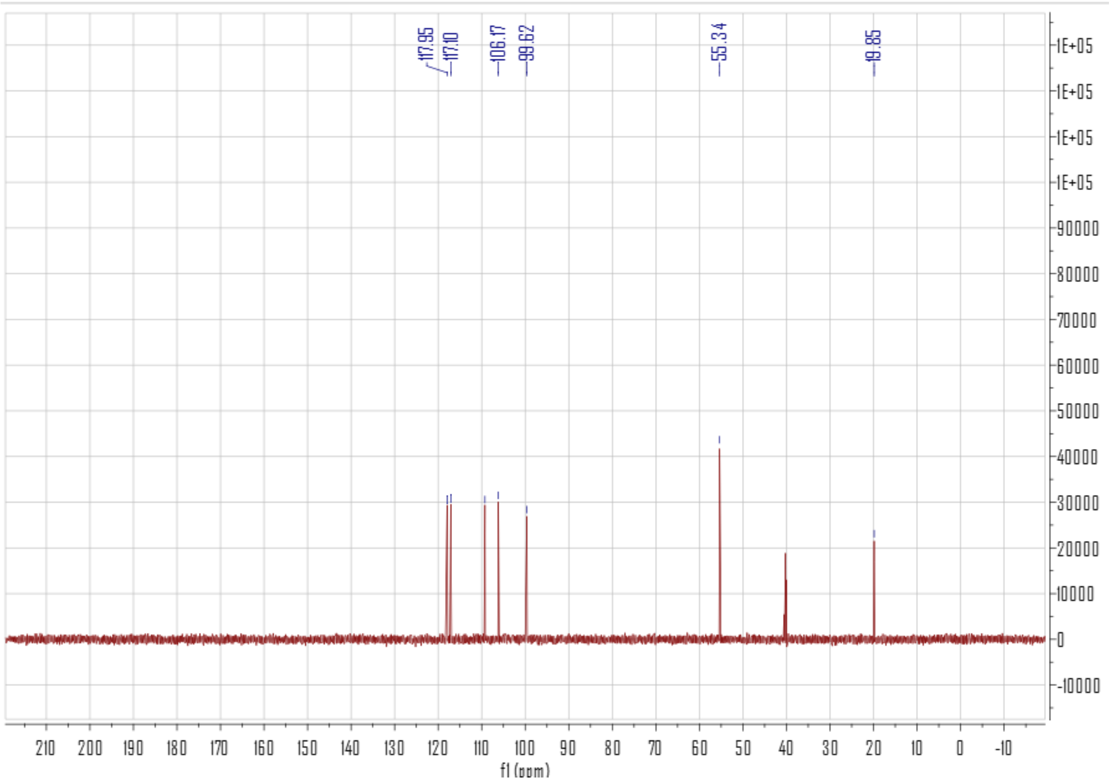

Figure S29. The ^{1}H NMR (500 MHz, DMSO-d_6) spectrum of 6 in DMSO

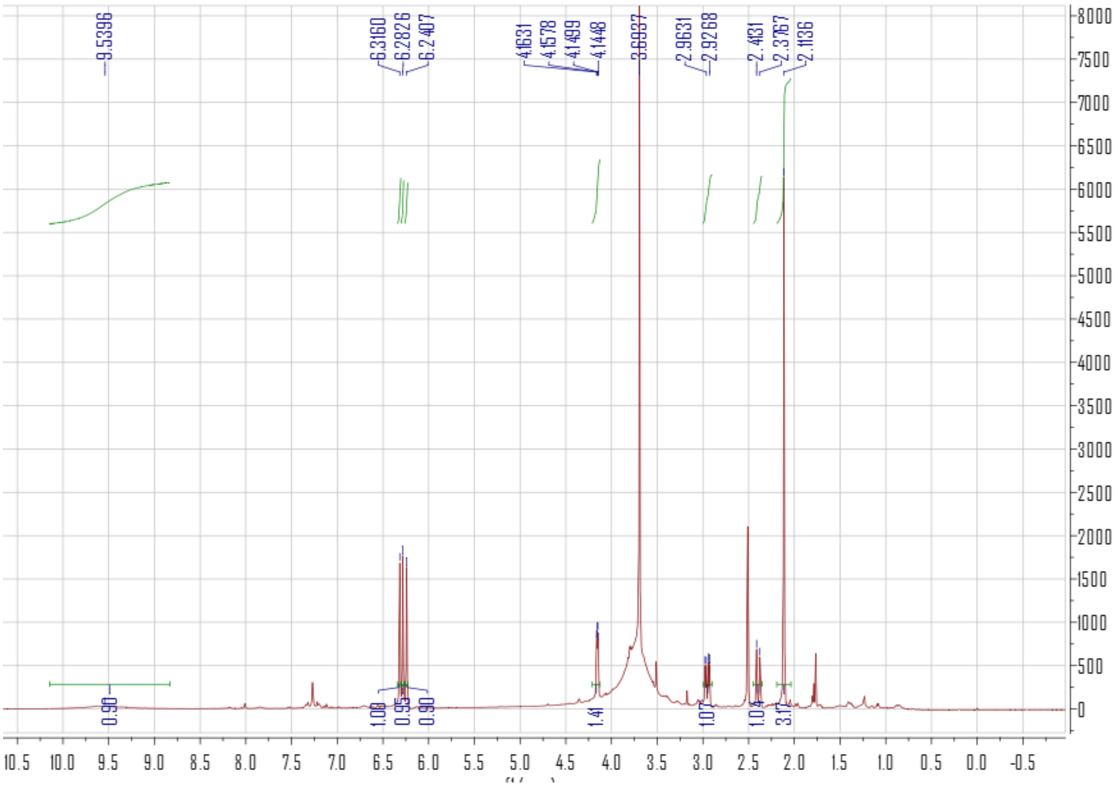

Figure S30. The ^{13}C NMR (125 MHz, DMSO-d_6) spectrum of 6

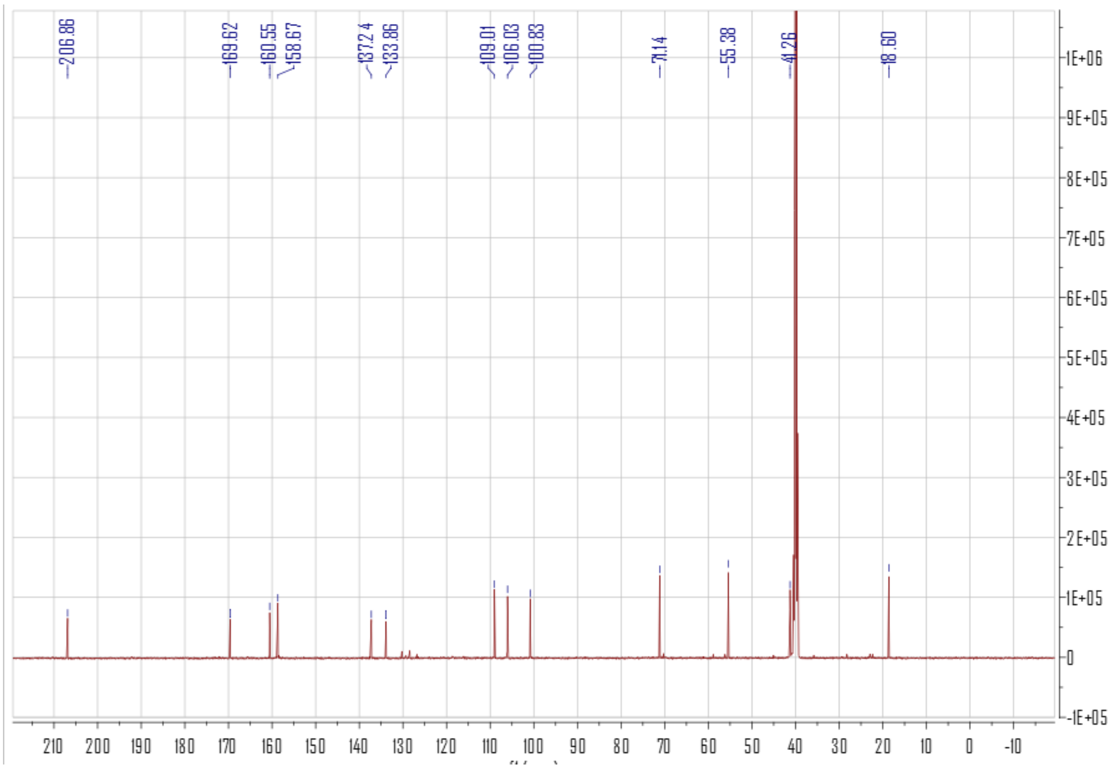

Figure S31. The DEPT-135 (125 MHz, DMSO-*d*$_6$) spectrum of 6

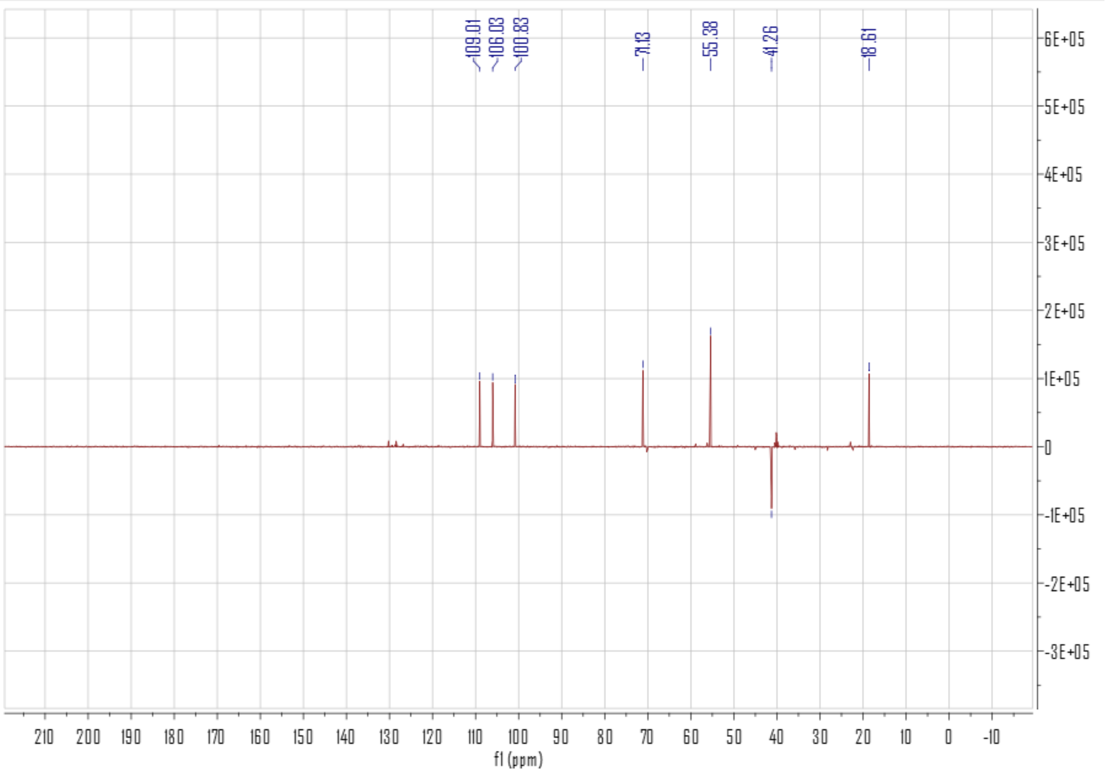

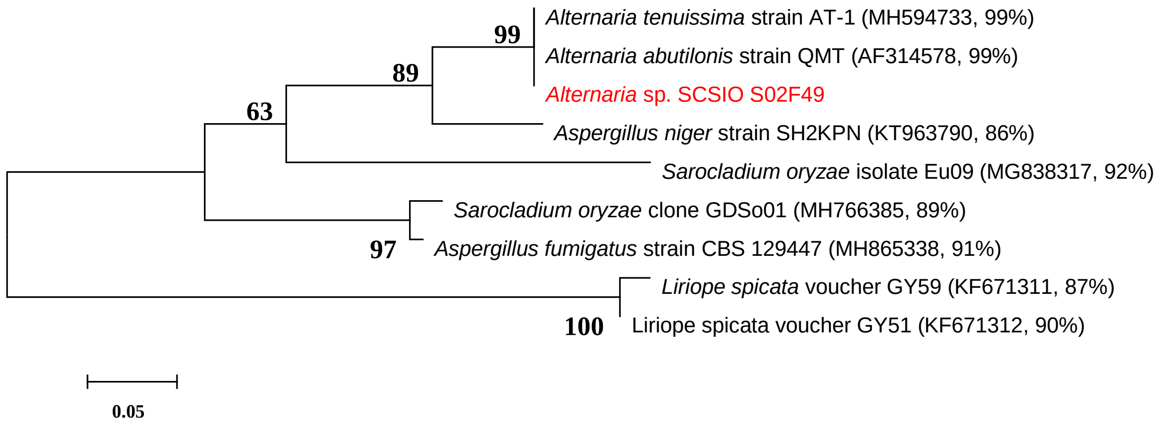

Figure S32. Phylogenetic trees of *Alternaria* sp. SCSIO S02F49
